# Simulation-based validation of a method to detect changes in SARS-CoV-2 reinfection risk

**DOI:** 10.1371/journal.pcbi.1012792

**Published:** 2025-02-03

**Authors:** Belinda Lombard, Harry Moultrie, Juliet R.C. Pulliam, Cari van Schalkwyk

**Affiliations:** 1 South African DSI-NRF Centre of Excellence in Epidemiological Modelling and Analysis (SACEMA), Stellenbosch University, Stellenbosch, South Africa; 2 Division of the National Health Laboratory Service, National Institute for Communicable Diseases, Johannesburg, South Africa; Northeastern University, UNITED STATES OF AMERICA

## Abstract

Given the high global seroprevalence of SARS-CoV-2, understanding the risk of reinfection has become increasingly important. Models developed to track trends in reinfection risk should be robust against possible biases arising from imperfect data observation processes. We performed simulation-based validation of an existing catalytic model designed to detect changes in the risk of reinfection by SARS-CoV-2. The catalytic model assumes the risk of reinfection is proportional to observed infections. Validation involved using simulated primary infections, consistent with the number of observed infections in South Africa. To assess the performance of the catalytic model, we simulated reinfection datasets that incorporated different processes that may bias inference, including imperfect observation and mortality. A Bayesian approach was used to fit the model to simulated data, assuming a negative binomial distribution around the expected number of reinfections, and model projections were compared to the simulated data using different magnitudes of change in reinfection risk. We assessed the model’s ability to accurately detect changes in reinfection risk when included in the simulations, as well as the occurrence of false positives when reinfection risk remained constant. The model parameters converged in most scenarios leading to model outputs aligning with anticipated outcomes. The model successfully detected changes in the risk of reinfection when such a change was introduced to the data. Low observation probabilities (10%) of both primary- and reinfections resulted in low numbers of observed cases from the simulated data and poor convergence. The model’s performance was assessed on simulated data representative of the South African SARS-CoV-2 epidemic, reflecting its timing of waves and outbreak magnitude. Model performance under similar scenarios may be different in settings with smaller epidemics (and therefore smaller numbers of reinfections). Ensuring model parameter convergence is essential to avoid false-positive detection of shifts in reinfection risk. While the model is robust in most scenarios of imperfect observation and mortality, further simulation-based validation for regions experiencing smaller outbreaks is recommended. Caution must be exercised in directly extrapolating results across different epidemiological contexts without additional validation efforts.

## Author summary

Most people have been infected with SARS-CoV-2, which increases interest in understanding the risk of reinfection. Our research focuses on the development of robust models that track reinfection trends, specifically emphasizing their resilience against biases from imperfect data observations. This study involves a simulation-based validation, which includes generating datasets under real-world scenarios of observation bias, to assess the reliability of an existing catalytic model designed to detect changes in the risk of SARS-CoV-2 reinfection in South Africa.

In our investigations, we found that the model parameters converged in most scenarios, aligning the model outputs with anticipated outcomes. Notably, the model successfully identified changes in reinfection risk when introduced to the data. However, challenges arose in scenarios that led to low numbers of observed cases, leading to poor parameter convergence.

This study underscores the significance of ensuring model parameter convergence to avoid false-positive detection of shifts in reinfection risk. While our model demonstrates robustness in scenarios of imperfect observation and mortality, additional simulation-based validation is recommended in regions with smaller outbreaks.

## Introduction

The COVID-19 pandemic has had catastrophic health, economic, and social impact, directly affecting billions of lives. As of July 2023, the pandemic had resulted in at least 6.9 million deaths globally [1]. Five major waves of infections were observed in South Africa. The first wave, driven by the original strain, peaked in mid-2020 and was followed by a second wave driven by the Beta variant towards the end of 2020. The Delta variant drove the third wave, in mid-2021, and the fourth and fifth waves were driven by the BA.1/ BA.2 and BA.4/ BA.5 Omicron sub-variants, at the end of 2021 and May 2022 [2]. These waves, coupled with vaccination efforts, have resulted in high levels of seroprevalence and relatively low numbers of reported infections since mid-2022 [3].

Reinfection with SARS-CoV-2 has emerged as a concern, due to waning immunity following infection and imperfect immunity, whereby prior infection does not provide full protection against reinfection [4]. Viral evolution also leads to the emergence of new variants, which may increase risk of reinfection [5].

Understanding the risk of reinfection by SARS-CoV-2 and potential future epidemics with other pathogens which do not result in lifelong immunity, has significance for both individual and public health. At the individual level, awareness of a high risk of reinfection might encourage individuals to take necessary precautions. In the public health context, understanding the risk of reinfection can help health officials make more informed decisions, potentially recommending increased practice of protective measures like hand sanitising and mask-wearing in public spaces, particularly if the reinfection risk is high.

Modelling studies have played a crucial role in understanding SARS-CoV-2 reinfection patterns. A Susceptible-Exposed-Asymptomatic-Infectious-Recovered (SEAIR) epidemic model that includes reinfections has been developed and applied to SARS-CoV-2 data in Pakistan, highlighting the importance of understanding reinfections in controlling disease spread [6]. Similarly, a Brazilian study utilised a more complex compartmental disease model, incorporating hospitalisation and deaths to assess the force of reinfections by the P.1 variant confirming that the P.1 variant significantly contributed to a surge in reinfections [7]. More recently, a study published in 2023 utilised real-world data to assess reinfection risks, particularly in the context of post-Omicron reinfections, further highlighting the evolving nature of SARS-CoV-2 reinfection dynamics and the importance of robust models to track these changes [8]. However, there is a lack of extensive validation studies that rigorously assess the robustness of these models under varying real-world conditions and biases.

A catalytic model was developed to monitor SARS-CoV-2 reinfection trends in South Africa, providing estimates for expected reinfections over time to detect population-level shifts in reinfection risks [9]. Using Monte-Carlo Markov Chains (MCMC), the model was set up as a null model by fitting to data on observed reinfections during the first two waves of SARS-CoV-2 in South Africa. The constant reinfection hazard coefficient estimated in the null model was used to project reinfection numbers during the two subsequent waves to monitor for divergence from the expectation under this model. Notably, the number of observed reinfections remained within the projection interval during the third (Delta) wave. However, in the fourth (Omicron BA.1/BA.2) wave, observed reinfections diverged from the model’s projections, indicating the Omicron variant’s potential for immune escape from prior infections [9].

Observed infection data, however, is not always fully representative of real-world patterns [10] due to factors such as undetected asymptomatic or mild cases, inaccessible testing centres, and variations in testing behaviour [11,12]. These influences, coupled with underreporting of SARS-CoV-2 infections and COVID-19 mortality, may distort the estimate of the population at risk for reinfection, which can possibly introduce misleading signals in reinfection risks [13,14].

In this study, we conduct simulation-based validation to evaluate the catalytic model’s performance and assess the robustness of the Omicron-related detection [9]. By incorporating different biases that represent real-world phenomena to simulated data, we determine whether the model reliably detects true shifts in reinfection risk, rather than signals introduced by data limitations. This approach helps confirm the model’s reliability for identifying genuine changes in reinfection risk and immune escape events.

## Materials and methods

### The catalytic model

The catalytic model assesses changes in reinfection risk by SARS-CoV-2 by accounting for the number of previously infected individuals and the changing infection risk through time [9]. Reinfections are defined as two positive tests at least 90 days apart, a period chosen to ensure that successive positive tests result from reinfection rather than prolonged viral shedding. Consequently, the model sets the risk of reinfection at zero for the first 90 days, and thereafter, it is proportional to the 7-day moving average of observed infections.

The probability of a positive test for SARS-CoV-2 by day *x* after *t* is given by the equation:


$$p\left(t,x\right)=1-e^{-\overset{i=x}{\underset{i=t+90}{\sum }}\lambda {Î}_{i}}$$

where *λ* is the reinfection hazard coefficient and ${Î}_{i}$ is the 7-day moving average of the total number of infections (both primary infections and reinfections) on day *i*.

The expected number of cases where the first positive test was on day *t* with a detected reinfection by day *x* is given by $I_{t}p\left(t,x\right)$, where $I_{t}$ is the number of putative primary infections reported on day t.

Thus, the expected number of reinfections by day *x* can be expressed as:


$$Y_{x}=\sum_{t=0}^{t=x}I_{t}p\left(t,x\right)$$

### Model fitting and projection

The catalytic null model, which assumes a constant reinfection hazard coefficient, can be fitted to the number of observed reinfections up until a defined fitting date.

In this process, two parameters were fitted using Metropolis-Hastings Monte Carlo Markov Chains (MCMC), the reinfection hazard coefficient (*λ*) and the negative binomial dispersion parameter (*κ*), assuming that the number of reinfections follows a negative binomial distribution. The first 4,000 of 10,000 iterations in each of four MCMC chains were discarded as burn-in.

To achieve a final joint posterior distribution of 1,600 parameter sets, we selected every 15^th^ sample from the joint distribution of the chains after excluding the burn-in. This approach ensures enough posterior samples to capture the parameter uncertainty effectively while maintaining computational efficiency. Each sample in the joint posterior distribution was used to simulate 100 stochastic realisations of expected daily reinfections. The stochastic realisations were used to obtain a 95% uncertainty interval for the fitting period, and a 95% credible interval for the ‘projection period’ (the time after the fitting date) under the null model.

### Simulation-based validation

We constructed a simulated dataset of primary infections representing a world in which all SARS-CoV-2 infections are observed, and no deaths occur, therefore every infected individual becomes eligible for reinfection after a 90-day period. The data was simulated, and all analyses conducted in the R Statistical Programming Language [version 4.3.1 (2023-06-16)] [15]. The full codebase and detailed documentation for reproducing all data and analyses are openly available at https://github.com/SACEMA/reinfectionsBelinda. The simulations were conducted on a cluster supercomputer leveraging 24 CPU cores to parallelise runs and improve computational efficiency.

**Fig 1:**
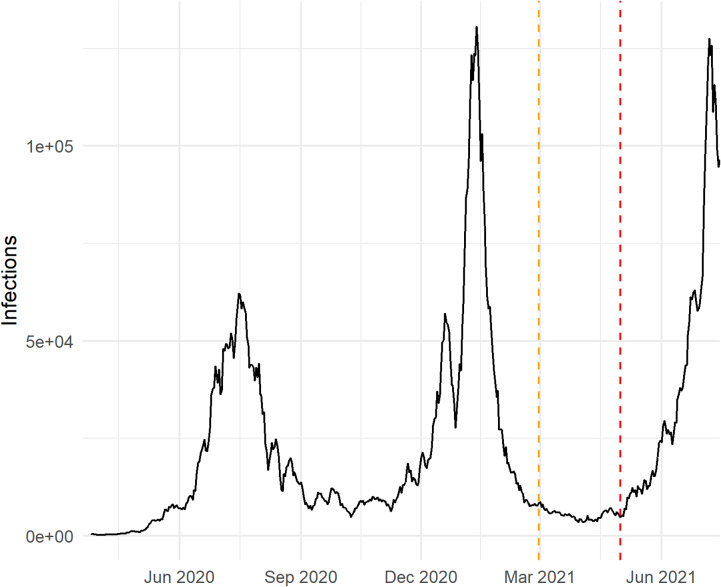
Simulated daily SARS-CoV-2 infections representing three distinct waves in South Africa. The simulated data is based on observed cases in South Africa up to 15 June 2021 by scaling the number of cases and adding noise to the number of infections to reflect variation in infection counts. The yellow dotted line represents the fitting date (28 February 2021), and the red dotted line represents the date after which an increase in reinfection risk is simulated (1 May 2021).

Fig 1 illustrates the simulated dataset representing the number of primary infections per day. This dataset of primary infections is based on the number of observed infections in South Africa through July 2021. Specifically, we generated a simulated time series of primary infections by taking the seven-day moving average of primary infections from the previously published South African data [16]. We increased the seven-day moving average by a factor of 5, then took a negative binomial sample around this mean with a shape parameter of $\frac{1}{\kappa }$, where κ≈0.27 was the median of the posterior sample from [9]. For dates at the beginning of the time series for which a seven-day moving average could not be calculated, the observed count was inflated by a factor of five and used as the mean for the negative binomial draw.

### The simulated scenarios

We utilised five scenarios to assess the model’s robustness and reliability of our chosen metric for detection of a change in reinfection risk. The first four scenarios depict an increasingly more realistic model world, with the fifth scenario considering a more complex description of varying observation probability as described in Table 1.

**Table 1:** Summary of each scenario used in the simulation-based validation, illustrating a progression towards more realistic data generation processes from Scenario A to D and Scenario E covering a scenario of varying observation probability without mortality.

Simulation	Description
**Scenario A**	All primary infections and reinfections observed, zero mortality
**Scenario B**	Imperfect observation for reinfections, zero mortality
**Scenario C**	Imperfect observations of reinfections and primary infections, zero mortality
**Scenario D**	Imperfect observations of primary infections and reinfections with mortality
**Scenario E**	Imperfect observation, with observation probabilities that change as a function of the number of infections, zero mortality

As part of the simulation-based validation, we sought to determine when a change in the hazard coefficient may be detected in the model, given the real-world evidence that certain variants carry higher risks of reinfection [9].

For data generation, a scale parameter was introduced after a ‘scale date’ (which we used as 1 May 2021) that is used to represent an Omicron-like wave, which varied from 1 (representing no change in reinfection risk) to 3 by steps of 0.1:


$$hz_{t}=\lambda \;.\;\;\sigma_{t}$$

where $hz_{t}$ is the modified hazard coefficient used to calculate reinfections on day t, *λ* is the reinfection hazard coefficient (obtained from the median of the posterior distribution of the fitted reinfection hazard coefficient in [9]) and $\sigma_{t}$ is a modifier on the hazard defined as


$$\sigma_{t}= \{\begin{array}{c} 1\;\;if\;\;t\leq 31\;April\;2021\\ \sigma \;if\;t>1\;May\;2021 \end{array}$$

We used *σ* to represent an increase in reinfection risk to represent the Omicron-like wave and varied σ≥1 in the different scenarios.

Detailed simulations for each scenario, including calculations and parameter adjustments, are described below.

### Scenario A: Perfect observation and no mortality

The baseline scenario assumes complete observation of all SARS-CoV-2 infections without mortality. This will determine the model’s ability to converge when all cases are observed and its ability to detect changes in the risk of reinfection with different magnitudes of these changes.

Reinfection is calculated as:


$$r_{t}=hz_{t}.i_{t}\;.\;e_{t}\;$$

where $hz_{t}$ is the modified reinfection hazard coefficient, $i_{t}\;$is the number of underlying primary infections on day *t* (Fig 1) and $e_{t}$ represents the number of people that are eligible for reinfection on day *t* and is calculated as follows:


$$e_{t}=\sum_{t=0}^{t-89}i_{t}-\sum_{t=90}^{t-1}r_{t}$$

The number of people eligible for reinfection, $e_{t}$, is calculated by subtracting the number of people who have already been reinfected with SARS-CoV-2 from those who had a primary infection at least 90 days ago, by that day.

The only parameter that is varied in this scenario is the scaling of the reinfection hazard $\left(\sigma \right)$. The data simulated for this scenario can be seen in S1 Fig.

### Scenario B: Imperfect observation of reinfections

Scenario B introduces the concept of imperfect observation of reinfections, reflecting real-world epidemiological limitations. By introducing this variable, we aim to evaluate the impact of imperfect observation of reinfections on the model’s robustness.

We use the binomial distribution to calculate ${\tilde{r}}_{t}$, the number of observed reinfections based on a varying observation probability, $P_{2}$, from 0.1 to 0.5 in increments of 0.1:


$${\tilde{r}}_{t}=Binomial\left(r_{t},\;P_{2}\right)$$

where $r_{t}$ is defined as in Scenario A. The observed number of people eligible for reinfection is represented by ${\tilde{e}}_{t}$ and is based on the number of observed reinfections, instead of the underlying reinfections. It is calculated as:


$${\tilde{e}}_{t}=\sum_{t=0}^{t-89}i_{t}-\sum_{t=90}^{t-1}\tilde{r}t$$

The data simulated for this scenario can be seen in S2 Fig.

### Scenario C: Imperfect observation of reinfections and primary infections

Expanding on Scenario B, this scenario also considers imperfect observation of both primary infections and reinfections, each with a specified observation probability. Three parameters are varied in this scenario: primary infections observation probability $\left(P_{1}\right)$, reinfections observation probability $\left(P_{2}\right)$ and the scale parameter $\left(\sigma \right)$.

Adjusting for imperfect observation, primary infections are calculated as:


$${\tilde{1}}_{t}=Binomial\left(i_{t},P_{1}\right)\;$$

where, $P_{1}$ denotes the observation probability for primary infections, which is varied in this scenario from 0.1 to 0.5 in increments of 0.1.

Only observed primary infections would be eligible for reinfection in a real-world dataset, therefore the number of underlying reinfections per day is calculated as follows:


$$r_{t}=hz_{t}.{\tilde{1}}_{t}.\;e_{t}$$

where $\;e_{t}$ is calculated as:


$$e{\;}_{t}=\sum_{t=0}^{t-89}\tilde{1}t-\sum_{t=90}^{t-1}r_{t}$$

Adding an observation probability to the number of reinfections like in Scenario B, the observed number of people eligible for reinfection is determined as follows:


$$\tilde{e}{\;}_{t}=\sum_{t=0}^{t-89}\tilde{1}t-\sum_{t=90}^{t-1}{\tilde{r}}_{t}$$

The data simulated for this scenario can be seen in S3 Fig.

### Scenario D: Imperfect observations of primary infections and reinfections with mortality

This scenario includes mortality among primary infections, which impacts the cohort susceptible to reinfection, giving a more refined perspective on SARS-CoV-2 transmission dynamics. The number of deaths resulting from observed primary infections is calculated as:


$$d_{t}=Binomial\left({\tilde{1}}_{t},\;d_{1}\right)\;$$

In this analysis, we varied the probability of dying ($d_{1}$) with values of 0.001, 0.01, and 0.05 representing a feasible range for infection related mortality to test the robustness of the method [3,17] The number of observed reinfections and primary infections are calculated as in Scenario C.

The number of people eligible for reinfection is adjusted by factoring in those who died from a primary infection and thus cannot be reinfected, calculated as:


$$e_{t}=\sum_{t=0}^{t-89}\tilde{1}t-\sum_{t=90}^{t-1}r_{t}-\sum_{t=0}^{t-89}d_{t}$$

The number of observed people who remain susceptible to reinfection is then calculated as:


$${\tilde{e}}_{t}=\sum_{t=0}^{t-89}\tilde{1}t-\sum_{t=90}^{t-1}{\tilde{r}}_{t}-\sum_{t=0}^{t-89}d_{t}$$

### Scenario E: Imperfect observation, with observation probabilities that change as a function of the number of infections

Reflecting a real-world setting, where potential changes in testing behaviour could be influenced by the perceived infection prevalence and/or the saturation of testing services during a surge, this scenario introduces dynamic observation probabilities ($P_{1}$ and $P_{2}$) as a function of the number of underlying infections. It tests the model’s adaptability to changes in testing behaviour.

We calculate the observation probabilities on day t as:


$$f\left(i_{t}\right)=min+\frac{max-min}{1+e^{s*\left(i_{t}-x_{m}\right)}}$$

**Fig 2:**
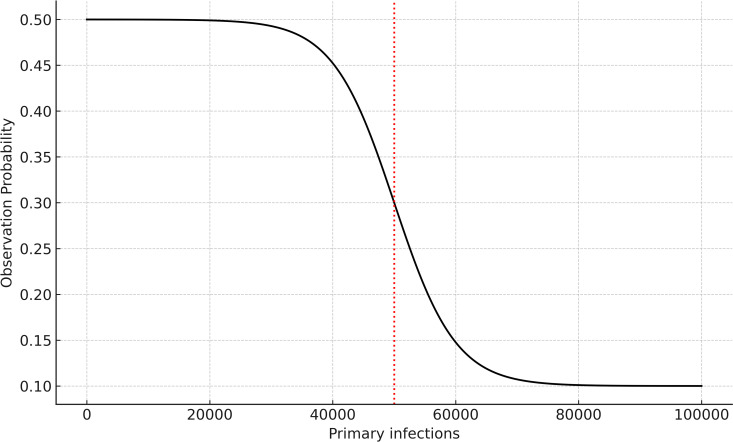
An graphical representation of the changing observation probabilities as the number of daily underlying primary infections increases. The observation probability decreases as infection counts rise, reflecting the declining likelihood of testing as the infection numbers grow. The steepness parameter, s, represents the speed of decline in observation probability with respect to primary infections, and the midpoint parameter, m, indicated with a red dotted line, represents the midpoint of the decline in observation probability.

In this equation, min and max are represented by the minimum and maximum observation probabilities respectively, *s* is the steepness parameter and $x_{m}$ is the ‘mid-point’. Fig 2 visually depicts f.

The maximum and minimum observation probabilities for reinfections ($P_{2}^{max}$ and $P_{2}^{min}$) are defined such that:

$P_{2}^{max}\geq P_{1}^{max}$ and


$$P_{2}^{min}\geq P_{1}^{min}$$

where $P_{1}^{max}$ and $P_{1}^{min}$ are the observation probabilities for primary infections, respectively.

We hypothesise that people who are reinfected are more likely to get tested for SARS-CoV-2 since they tested for their primary infections. We excluded parameter sets where $P_{1}^{min}=P_{1}^{max}$ and $P_{2}^{min}=P_{2}^{max}$ as this would replicate Scenario C.

Multiple parameters are varied in this scenario: the maximum and minimum observation probability for primary infections ($P_{1}^{max}$ and $P_{1}^{min}$), the maximum and minimum observation probability for reinfections ($P_{2}^{max}$and $P_{2}^{min}$), the steepness of the function (*s*), the midpoint of the function $\left(x_{m}\right)$, and the scale (*σ*).

Each of the varying parameters in Scenario E are described in Table 2. The values for *σ* were selected to represent both a modest increase in reinfection risk (σ = 1.2) and a more substantial increase. We observed that the increase in reinfection risk was consistently detectable when σ > 1.5 indicating that extending the analysis to these higher values would be redundant. Based on the estimated proportion of observed primary infection cases in 2022 in South Africa and presumed higher testing probabilities in settings with better access to testing, we set the values of $P_{1}^{min}$ and $P_{1}^{max}$ to range between 0.1 and 0.5 [3,18]. We didn’t consider values below 0.1, as we have seen that the model parameters fail to converge at observation probabilities lower than 0.1. Three different mid-point values were considered representing the number of underlying primary infections during the upward trajectory of each simulated wave (Fig 1).

**Table 2:** Parameters varied in Scenario E with corresponding values.

Parameter	Values
Scale (*σ*)	{1; 1.2; 1.5}
Maximum observation probability for primary infections ($P_{1}^{max}$)	$\left\{0.2;\;0.3;\;0.4;\;0.5\right\}\;$
Minimum observation probability for primary infections ($P_{1}^{min}$)	$\left\{0.1;\;0.2;\;0.3;\;0.4;\;0.5\right\}$ such that $P_{1}^{min}$<$P_{1}^{max}$
Maximum observation probability for reinfections ($P_{2}^{max}$)	{0.2; 0.3; 0.4; 0.5} such that $P_{2}^{max}\geq P_{1}^{max}$
Minimum observation probability for reinfections ($P_{2}^{min}$)	$\left\{0.1;\;0.2;\;0.3;\;0.4;\;0.5\right\}$ such that $P_{2}^{min}<P_{2}^{max}$ and$P_{2}^{min}\geq P_{1}^{min}$
Steepness (*s*)	$\left\{0.00005;0.0001\right\}\;$
Midpoint $(x_{m}$)	$\left\{30000;40000;50000\right\}$

### Evaluating model performance

We evaluated the performance of the catalytic model across Scenarios A to E, by simulating data specific to each scenario’s parameters and applying the model fitting and projection process to estimate a 95% projection interval for daily reinfections.

The fitting and projection process was repeated 20 times per scenario, each with different seed values affecting the data generation process (binomial draws), the MCMC fitting procedure and the model projection process.

We assessed parameter convergence and model fit during the fitting period, then applied a set of metrics to measure the impact of different scenario definitions on the model performance. These metrics included: assessing the first cluster of reinfections above the projection interval during the projection period, determining the proportion of infections above the projection interval during the projection period, and evaluating the specificity of detecting simulated changes in reinfection risk.

### Parameter convergence

Parameter convergence for the reinfection hazard coefficient (λ) and the negative binomial dispersion parameter (κ) was measured using Gelman-Rubin diagnostics, a ratio that compares the between-chain and within-chain variances. A value below 1.1 indicates that the parameter converged [19]. To validate the robustness of the Gelman-Rubin diagnostic, we also used the Geweke diagnostic to assess convergence of Scenario D (S1 Text) [20].

### Exclusion of non-converging runs or poor model fit

Non-convergence of *κ* leads to narrow projection bands, resulting in more observed reinfections falling outside the projection interval and possible incorrect conclusions regarding trends in the risk of reinfection. Similarly, it is crucial for *λ* to converge to ensure reliable predictions. We therefore excluded runs where either λ or *κ* did not converge from our analysis (when the Gelman-Rubin convergence diagnostic for that parameter was above 1.1). Additionally, runs showing clusters of five consecutive values above or ten consecutive values below the 95% projection interval for the 7-day moving average of reinfections during the fitting period (before the fitting date, 28 February 2021) were excluded. Such a cluster implies that the model inaccurately represents the patterns seen in the simulated data.

### Timing of first cluster of reinfections above the projection interval

The timing of a first cluster of five consecutive points (days) above the projection interval of the 7-day moving average $\left(D_{first}\right)$ is a metric that can be used for detection of a change in the hazard coefficient. This approach balances the need for real-time detection with specificity - a five-day cluster enables timely insights into changes in reinfection risk while managing the potential for false positives. The presence of such a cluster indicates a possible increase in reinfection risk. In our simulated data, where we have introduced an increasing hazard coefficient (σ>1), we used $D_{first}\;$to assess the magnitude of change required for our approach to detect it. As a summary metric, we calculated the median of $D_{first}$ after excluding non-converging runs and runs with clusters of reinfections outside the projection interval in the fitting period, as described above.

### Proportion of infections above the projection interval

In runs with no increase in the risk of reinfection, we expect that the number of daily observed reinfections exceeding the 95% projection interval would be less than 2.5% of the days in the projection period. If this proportion exceeds 2.5%, it indicates either a successfully detected increase (when σ>1) or a false positive detection (when σ=1). As a summary metric, we calculated the median of this proportion across runs that were not excluded by the criterion described above.

Measuring the proportion of infections above the projection interval helped us to assess the magnitude of change in reinfection risk likely to be detected by the method and the effect of the potential biases we examined. It is important to note that this measurement does not enable us to assess real-time performance of the method but is a general indicator of robustness.

### Specificity

Specificity is a proportion used to measure the approach’s reliability when there is no change in the risk of reinfection. We measured the specificity for each scenario definition where σ=1 by calculating it across the 20 runs as


$$specificity=1-\frac{Number\;of\;runs\;where\;D_{first\;}exists\;\&\;run\;not\;excluded}{Number\;of\;runs\;not\;excluded}$$

High specificity indicates that false positive detections of a change in reinfection risk are unlikely.

## Results

### Parameter convergence

Across Scenarios A to D, the Gelman-Rubin convergence diagnostics were below 1.1 for *λ* (the reinfection hazard coefficient) and *κ* (the negative binomial dispersion parameter) across most scenario definitions, which indicates convergence. (S4 and S5 Figs). However, at low observation probabilities (for example, at $P_{1}=0.1$ and $P_{2}=0.2$), the proportion of runs that converged ranged between 0.65 and 0.85 across Scenarios. At the lower extremes for $P_{1}$ and $P_{2}$ ($P_{1}=0.1$ and $P_{2}=0.1$), the proportion of runs that converged ranged between 0.05 and 0.3 across Scenarios, with an increase observed as the probability of dying from a primary infection ($d_{t}$) decreased (S4 and S5 Figs).

In Scenario E, with low observation probabilities ($P_{1}^{min}=P_{2}^{min}=0.1$, $P_{1}^{max}=P_{2}^{max}=0.2)$, low steepness of the function, and a low midpoint, most of the runs did not converge (S7 and S8 Figs). These scenarios corresponded to relatively few observed primary infections and consequently, few generated reinfections. At higher values of $P_{1}^{min}$, $\;P_{1}^{max}$, $P_{2}^{min}\;$and $\;P_{2}^{max}$, more than 75% of runs converged (S9 and S10 Figs).

As shown in the S6 Fig, using the Geweke diagnostic yielded similar results, supporting the convergence trends observed with the Gelman-Rubin diagnostic.

### Exclusion of non-converging runs or poor model fit

The number of runs excluded per run can be seen in Table 3.

**Table 3:** Summary of runs excluded in each Scenario. In all the summarised runs, σ=1. The values in brackets indicate the proportion of runs excluded.

Scenario	Number of runs	Excluded due to non-convergence	Excluded due to cluster outside projection interval during fitting period*
A	20	0 (0.0)	0 (0.0)
B	100	0 (0.0)	2 (0.2)
C	500	25 (0.05)	38 (0.08)
D	1,500	81 (0.054)	107 (0.075)
E	6,000	83 (0.014)	3,087 (0.522)

* The denominator used to calculate the proportion excludes runs that were excluded due to non-convergence.

In Scenarios C and D, the majority of the runs that were excluded was when $P_{1}<0.2$ and $P_{2}\leq 0.2$, (low values of observation). For the rest of the values of $P_{1}$ and $P_{2}$ (when $P_{1}>0.1$ and $P_{2}>0.2$), at most two of 20 was excluded due to non-convergence.

After excluding non-converging runs in Scenario E, 1,386 of the runs were excluded due to clusters of five consecutive points above the projection interval during the fitting period and a further 1,701 runs were excluded due to clusters of ten consecutive points falling below the projection interval during the fitting period. One such scenario definition can be seen in S11 Fig, where a cluster of 10 consecutive observed reinfections was below the projection interval during the fitting period. In these excluded runs, the simulated data did not match observed trends in South African data, where peaks in primary infections and reinfections were temporally correlated.

### First cluster of reinfections above the projection interval

**Fig 3:**
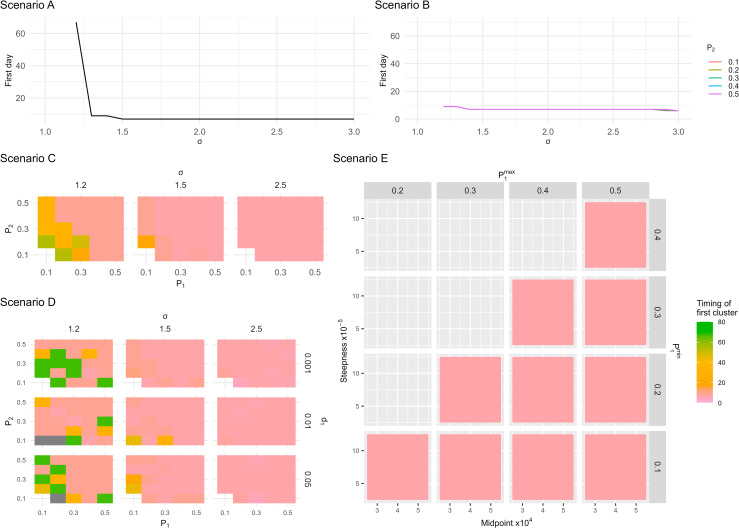
Timing of the first five consecutive points above the projection interval after an increase in reinfection risk has occurred. In Scenario E, σ = 1.5 and $P_{2}^{min}$ and $P_{2}^{max}$ are 0.4 and 0.5 respectively. Plots for other Scenario E definitions are shown in S12 Fig. The gaps in Scenario C and Scenario D represent scenario definitions where results of all runs were excluded because of non-convergence or a cluster outside the projection interval during the fitting period.

The timing of the first cluster of reinfections above the projection interval indicate effective detection of reinfection risk changes across all scenarios. In Fig 3, this timing is shown for all the scenarios, with visual representations indicating how early the model identifies increased reinfection risks.

In Scenarios A through D the median of $D_{first}$ is 7 days when σ≥1.5, indicating the change in reinfection risk is detected very soon after it is introduced in the data. In Scenarios C and D, when σ=1.2, the median of $D_{first}$ over the 20 runs was slightly higher for most scenario definitions, but the change in risk of reinfection was detected for all non-excluded runs. Generally, lower values of $P_{1}$ and $P_{2}$ were associated with slightly higher median values of$\;D_{first}$. In Scenario D, when the probability of mortality after experiencing an observed primary infection$,\;\;d_{1}$, is lower, the median $D_{first}$ was also slightly higher.

In Scenario E, when σ>1.2, the change in reinfection risk was detected for all non-excluded runs. However, when the number of observed cases (primary infections and reinfections) was lower, the median $D_{first}$ was higher (extending to around 40 days, as shown in S12 Fig).

### Proportion of infections above projection interval

**Fig 4:**
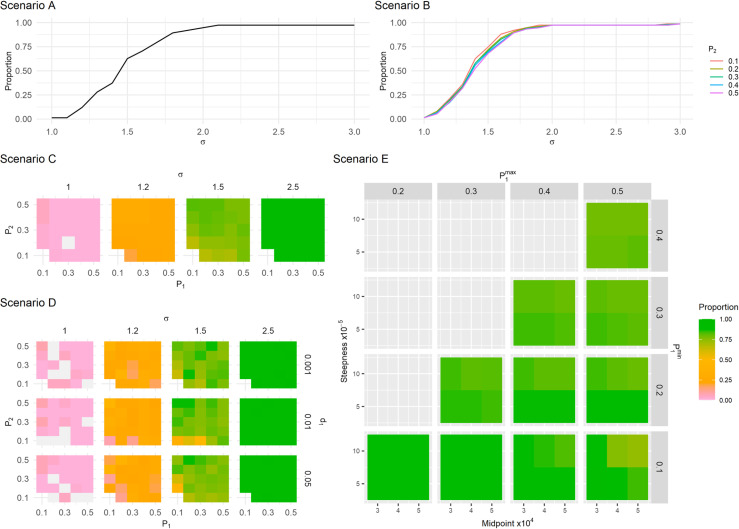
Median proportion of estimated reinfections above the projection interval following an increase in reinfection risk. The figure illustrates the relationship between the increase in risk (σ) and the median proportion of points above the interval across Scenarios A to E. The gaps in Scenario C and D represent scenario definitions where results from all runs were excluded. Scenario E further highlights the variability in the proportion of points above the interval depending on parameter combinations of steepness and midpoint values.

In Scenarios A to E, the proportion of reinfections above the projection interval gradually increased as the proportional increase in risk of reinfection (*σ*) increased, stabilising at around 0.973, as visually depicted in Fig 4, indicating effective detection of heightened reinfection risks across all scenarios.

In Scenario C, at σ=1.5 (representing a 50% increase in the reinfection hazard coefficient), the proportion of points outside the projection interval was higher when more infections are observed (Fig 4). Even at low observation probabilities, an increase in reinfection risk was still detected ($D_{first}$ still existed). For instance, when $P_{1}=0.1$ and $P_{2}=0.2$, the median proportion of points above the projection interval was 0.67 whereas the proportion was closer to 0.8 in scenarios where $P_{1}$ and $P_{2}$ was higher. For substantial increases in the reinfection hazard coefficient (σ≥1.7), the proportion of points above the projection interval was 0.973 (S13 Fig).

The values used for mortality in Scenario D had a minimal effect on the proportion of points outside the projection interval (Fig 4D).

In Scenario E, the median proportion of points above the projection interval varied from 49% to 95% when σ=1.5 (Fig 4). The median proportion was lower at higher values of steepness for most scenario definitions, and at the higher value of midpoint values (S14 Fig).

### Specificity

The specificity was 1 across all scenario definitions in Scenarios A and B. In Scenario C (S15 Fig), where we considered fixed $P_{1}$ and $P_{2}$, and Scenario D (S16 Fig), where we introduced mortality, the specificity was mostly above 0.95. However, in scenarios where $P_{1}=0.1$ and the difference between $P_{1}$ and $P_{2}$ was substantial, such as $P_{1}=0.1$ and $P_{2}=0.5$, the specificity dropped to 0.75. In both Scenarios C and D, the specificity remained above 0.75.

In Scenario E, the specificity approached 1 when a larger number of cases were observed (i.e., higher values of $P_{2}^{min}$, $P_{2}^{max},\;P_{1}^{min}$, $P_{1}^{max}$, the midpoint, and steepness) (see S18 Fig). Conversely, when fewer cases were observed, the specificity decreased. For instance, when $P_{2}^{min}$ and $P_{2}^{max}$ were 0.2 and 0.3 respectively, the specificity ranged from 0.33 to 0.91. Higher specificity values were observed when $P_{1}^{min}$ and $P_{1}^{max}$ were higher, and the midpoint of the function was greater, indicating more cases were observed, as can been seen in S18 Fig. When considering the runs where false positive increases in the reinfection risk were detected, most runs had a cluster of five consecutive observed reinfections below the interval during the fitting period, suggesting that the model did not align well with the trends in the data during the fitting period, yet these runs did not meet our exclusion criteria.

## Discussion

In this study we performed simulation-based validation on a method used for real-time monitoring of SARS-CoV-2 reinfections to detect changes in the risk of reinfection [9]. The model parameters converged well under various observation biases and the model is robust when dealing with changes in observation probability for reinfections. The model showed strong parameter convergence, indicating reliable projection interval simulation, particularly when patterns seen in the simulated data were well represented during the fitting procedure.

Low observation probabilities in all Scenarios (0.1) and parameter combinations in Scenario E causing low numbers of observed reinfections, impacted negative binomial dispersion parameter convergence, due to a lack of data to properly inform this parameter. This finding aligns with Pulliam *et al*. where the dispersion parameter did not converge over the short timeframe when fitting South African data over the first wave [21], underscoring the need for sufficient data to accurately inform parameters.

We measured when the first cluster of five observed reinfections fell above the projection interval to understand how soon the approach detects changes in the reinfection risk. In most scenarios, increases in reinfection risk as low as 20% was detected, and increases above 50% were detected soon after their introduction in the underlying data, highlighting the method’s sensitivity. However, in scenarios with fewer observed infections and reinfections, increased model parameter uncertainty caused slight delays in detecting an increase in reinfection risk.

Furthermore, when the observation probabilities were varied as a function of underlying primary infections, the proportion of observed reinfections above the projection interval for a given magnitude of increase in the reinfection hazard coefficient remained consistent despite changes in the function’s parameters. For instance, when reinfection risk increased by 50%, more than half (above 0.5) of the observed reinfections fell above the projection interval, indicating that the method is sensitive to increases in the reinfection risk.

We also evaluated specificity, measuring the proportion of scenarios where no increase in reinfection risk was detected (i.e., there were no stretches of five consecutive points above the projection interval), given that no such increase was present in the simulated data (where σ = 1). In scenarios where all primary infections were observed (Scenarios A and B), there were no false positive detections of changes in reinfection risk. However, when observation probabilities for primary infections were included alongside reinfections, there were some runs where false increases in the risk of reinfections were detected, particularly when the difference between observation probabilities for primary infections and reinfections was high (>0.3).

When observation probabilities were calculated as a function of the number of underlying primary infections, few false positives were detected when the number of observed cases were high. However, when the infections and reinfections observed are lower, especially when the observed reinfections did not fall well within the projection interval during the fitting period (i.e., there are large clusters below or above the projection interval), we advise more careful interpretation of apparent changes in the risk of reinfection. In such cases, criteria to detect an increase in the risk of reinfection could, for example, be extended to having a cluster of ten consecutive days above the projection interval during the projection period instead of five.

Changes in mortality had minimal impact on model performance, supporting its applicability across fluctuating mortality rates.

### Strengths and limitations

A major strength of this study is that we investigated the robustness of the model under different assumptions of observation probabilities that could occur in the real-world, enhancing the model’s practical applicability. We determined that the model outcome is not sensitive to changes in mortality rates which could be influenced in the real world by factors such as healthcare capacity, treatment effectiveness, and vaccination campaigns.

However, the study’s timeframe is a limitation, as ideally, this type of simulation-based validation should occur concurrently with real-time monitoring during an outbreak response.

Additionally, the simulated dataset used in the simulation-based validation is based on the situation in South Africa; thus, the findings may not be applicable to countries with significantly smaller populations, limited testing, or extensive vaccination coverage, resulting in lower numbers of observed infections and reinfections.

Lastly, the simulation-based validation did not consider waning natural immunity as a potential reason for an increase in the risk of reinfection. The method focuses on detecting a population-level increase in the reinfection risk but does not assign a mechanism to the detected increase; interpretation of the drivers of a change in reinfection risk requires triangulation with other data sources. That said, whilst there is evidence of waning natural immunity of SARS-CoV-2 [22], analysis of reinfection trends in South Africa was conducted from January 2021 through November 2022, with the only detected change in reinfection risk being associated with the emergence of the Omicron variant. This finding suggests that the dynamics of waning immunity for SARS-CoV-2 may not produce population-level increases in reinfection risk that are detectable using this method.

### Directions for future work

Further validation with data representative of different countries, population sizes, and vaccination histories is necessary to ensure its broader applicability. Understanding the impact of vaccination on reinfection risk and modification of the method for high vaccination coverage contexts are crucial next steps. Additionally, validation should be performed for other approaches to detecting changes in the risk of reinfection, like the Pulliam *et al*.’ approach which estimated time-varying infection and reinfection hazards [9]. Future work should also explore the role of waning immunity in population-level shifts in reinfection risk.

## Conclusions

Simulation-based validation demonstrates the method’s robust performance across imperfect observation and mortality scenarios. Specifically, model parameter convergence and good fit during the fitting period should be prerequisites when using the model to detect real-time increases in population-level reinfection risk. Although continued validation under different epidemiological contexts is necessary, the simulation-based validation enhances the catalytic model’s applicability in different real-world scenarios.

## Supporting information

S1 Text Geweke diagnostic. Additional information about the geweke diagnostic explored.
(DOCX)

S1 FigSimulated underlying primary infections with reinfections at different values of *σ.*The plot represents Scenario A, with figure A showing the simulated primary infections with perfect observation and no mortality, and B showing the observed reinfections with different values of σ used as input in Scenario A.(PNG)

S2 FigObserved reinfections for different observation probabilities for reinfections, $P_{2}$ , in Scenario B (no change in reinfection risk) and σ=1 .Scenario B has imperfect observation of reinfections.(PNG)

S3 FigThe observed primary infections and reinfections for Scenario C.A shows the number of observed primary infections for different values of $P_{1}$ and B shows the observed reinfections for different values of $P_{2}$ shown at the top of each grid. Each line depicts another value of$P_{1}.$(PNG)

S4 FigProportion of runs in Scenario C where both λ and κ converged.Here we introduced observation probabilities for primary infections and reinfections ($P_{1}$ and $P_{2}\;$respectively).(PNG)

S5 FigProportion of runs in Scenario D where both λ and κ converged.Here we added observation probabilities for primary infections, reinfections and we included mortality ($P_{1}$, $P_{2}$ and $d_{1}$ respectively).(PNG)

S6 Fig Convergence diagnostics using Geweke diagnostics for scenario D, showing the proportion of runs in Scenario D where both λ and κ converged. The Geweke diagnostic measures convergence by comparing the means of the first and last portions of a single Markov chain; if the Z-score is close to zero, it suggests convergence.
(PNG)

S7 FigThe proportion of runs that converged for Scenario E where $P_{2}^{min\;}=0.1\;$ and $P_{2}^{max}$ indicated at the top of each grid.(PNG)

S8 FigThe proportion of runs that converged for Scenario E where $P_{2}^{min\;}=0.2$ and $P_{2}^{max}\;$ indicated at the top of each grid.(PNG)

S9 FigThe proportion of runs that converged for Scenario E where $P_{2}^{min\;}=0.3$ and $P_{2}^{max}$ indicated at the top of each grid.(PNG)

S10 FigThe proportion of runs that converged for Scenario E where $P_{2}^{min\;}=0.4$ and $\;P_{2}^{max}$ indicated at the top of each grid.(PNG)

S11 Fig An instance of an ‘unrealistic’ time series where the model faced challenges in fitting the simulated reinfection data. During the fitting period preceding the dotted red line, the observed reinfections (depicted by the solid red line) consistently fell below the projection interval in January. In this particular scenario, the function determining the observation probability had a low midpoint of 30,000, minimal observation probabilities for primary- and reinfections set at 0.1 and 0.4, respectively, and a low steepness factor of 0.00005.
(PNG)

S12 Fig Plot showing the median of the timing of the first cluster of five days where the reinfections fell above the projection interval after the introduction of the scale (σ) for Scenario E. In A, the minimum and maximum observation probabilities for reinfections are 0.1 and 0.2. In B, the minimum and maximum observation probabilities for reinfections are 0.2 and 0.3. In C, the minimum and maximum observation probabilities are 0.3 and 0.4. The introduced scales (σ) are indicated at the top.
(PNG)

S13 Fig Plot showing the median of the proportion of points above the projection interval for Scenario C for different values σ. (PNG)

S14 Fig Plot showing the median of the proportion of points above the projection interval for Scenario E. In A, the minimum and maximum observation probabilities for reinfections are 0.1 and 0.2. In B, the minimum and maximum observation probabilities for reinfections are 0.2 and 0.3. In C, the minimum and maximum observation probabilities are 0.3 and 0.4. The introduced scales (σ) are indicated at the top.
(PNG)

S15 FigSpecificity ( σ=1 ) of Scenario C, over 20 runs with different fixed values of primary infections and reinfections observation probabilities.The numbers in the grid are the number of runs where both λ and κ converged and a cluster of five consecutive points above or 10 consecutive points below the projection interval during the fitting period does not exist. The specificity is measured as the number of those runs where $D_{first}$ does not exist, i.e., no false positive detection of a change in reinfection risk was observed.(PNG)

S16 FigSpecificity ( σ=1 ) of Scenario D over 20 runs with different fixed values of primary infections and reinfections observation probabilities where mortality is considered.The numbers in the grid are the number of runs where both λ and κ converged and a cluster of five consecutive points above or 10 consecutive points below the projection interval during the fitting period does not exist. The specificity is measured as the number of those runs where $D_{first}$ does not exist, i.e., no false positive detection of a change in reinfection risk was observed.(PNG)

S17 FigSpecificity ( σ=1 ) of Scenario E over 20 runs when $P_{2}^{min\;}=0.4$ and $P_{2}^{max}=0.5$ .The numbers in the grid are the number of runs where both λ and κ converged and a cluster of five consecutive points above or 10 consecutive points below the projection interval during the fitting period does not exist. The specificity is measured as the number of those runs where D_first does not exist, i.e., no false positive detection of a change in reinfection risk was observed.(PNG)

S18 FigSpecificity ( σ=1 ) of Scenario E over 20 runs when $\;P_{2}^{min\;}=0.2$ and $P_{2}^{max}=0.3$ .The numbers in the grid are the number of runs where both λ and κ converged and a cluster of five consecutive points above or 10 consecutive points below the projection interval during the fitting period does not exist. The specificity is measured as the number of those runs where D_first does not exist, i.e., no false positive detection of a change in reinfection risk was observed.(PNG)
