# Supplementary material for: Simulation-based validation of a method to detect changes in SARS-CoV-2 reinfection risk
Source: PLoS Comput Biol. 2025 Feb 3;21(2):e1012792. doi: 10.1371/journal.pcbi.1012792 (PMC11801736; doi:10.1371/journal.pcbi.1012792)
Supplement: S1 Text — Additional information about the geweke diagnostic explored. (DOCX) [file pcbi.1012792.s001.docx]

### Additional information: Geweke diagnostic

In addition to Gelman-Rubin convergence diagnostics, we explored an additional diagnostic, the Geweke diagnostic, specifically for Scenario D, to compare it with the results obtained using the Gelman-Rubin diagnostic. The Geweke diagnostic measures convergence as a function of the difference in the means of the first 10% and last 50% of a single Markov chain. If the difference between the two-sample means divided by its estimated standard error is close to zero (-1.96<Z<1.96), it suggests convergence. For each run, the convergence was taken as the mean Z-score of the four chains.
