# Supplementary figures and images for: Simulation-based validation of a method to detect changes in SARS-CoV-2 reinfection risk
Source: PLoS Comput Biol. 2025 Feb 3;21(2):e1012792. doi: 10.1371/journal.pcbi.1012792 (PMC11801736; doi:10.1371/journal.pcbi.1012792)

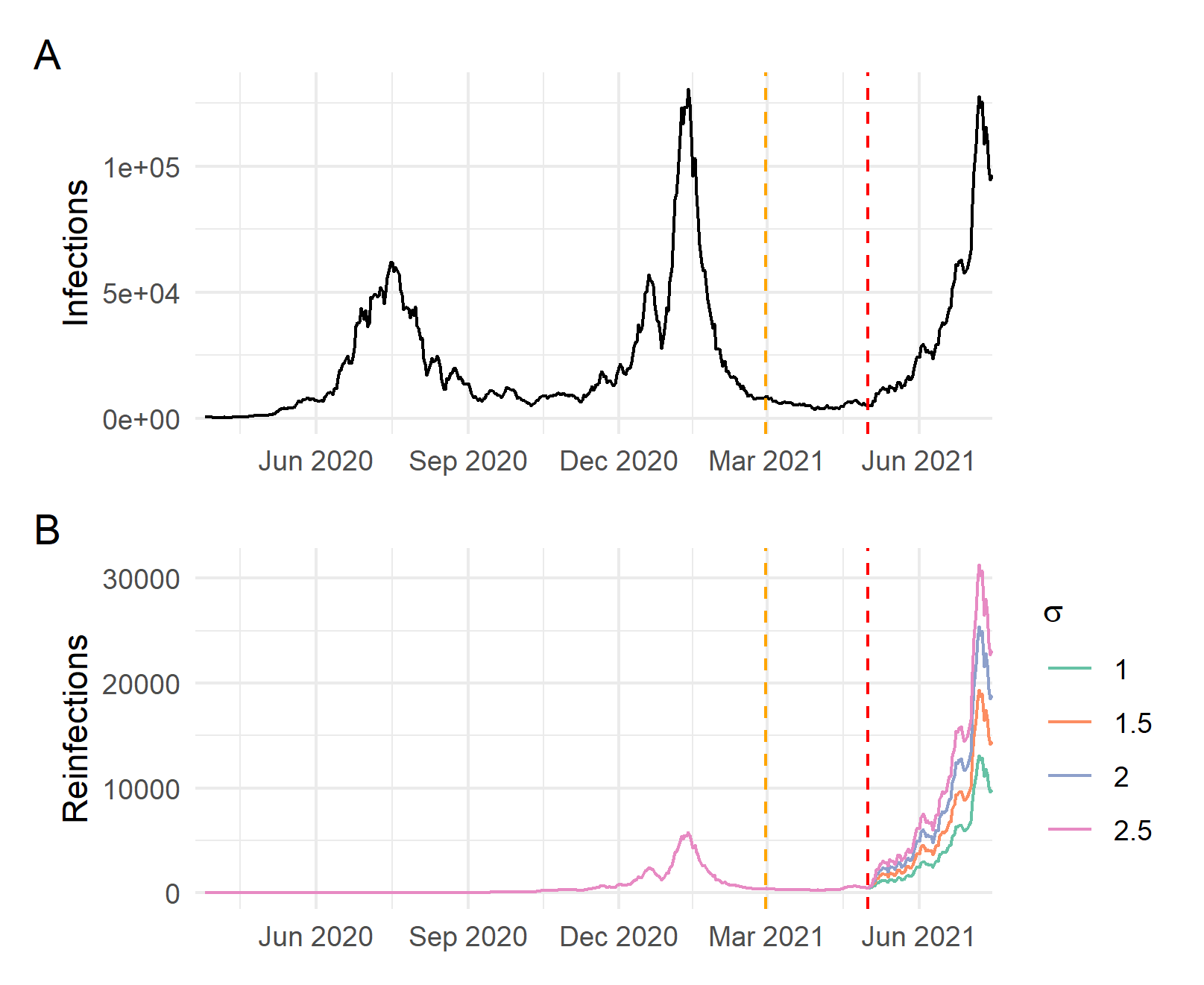

Supplement: S1 Fig — The plot represents Scenario A, with figure A showing the simulated primary infections with perfect observation and no mortality, and B showing the observed reinfections with different values of σ used as input in Scenario A. (PNG) [file pcbi.1012792.s002.png]

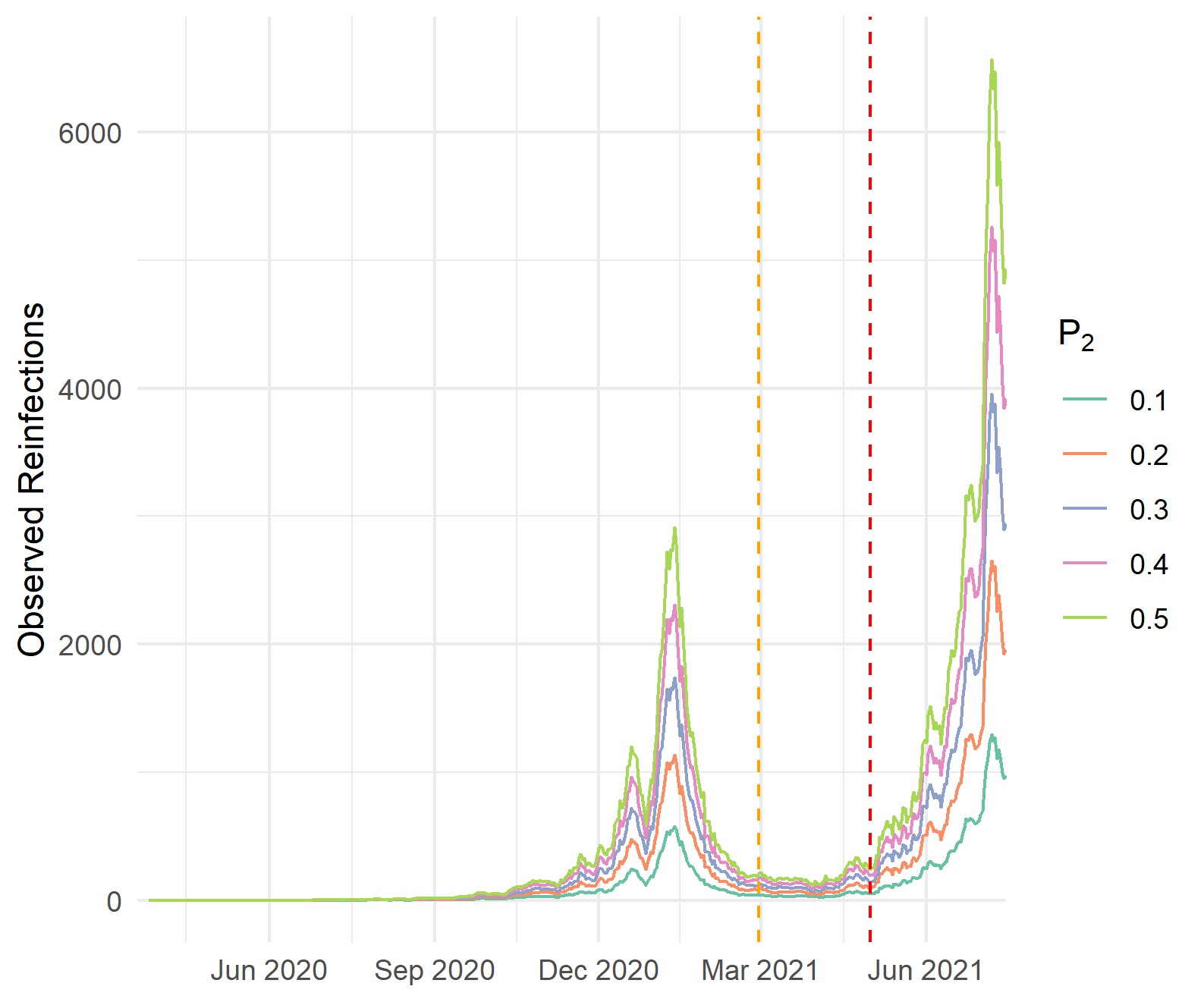

Supplement: S2 Fig — Scenario B has imperfect observation of reinfections. (PNG) [file pcbi.1012792.s003.png]

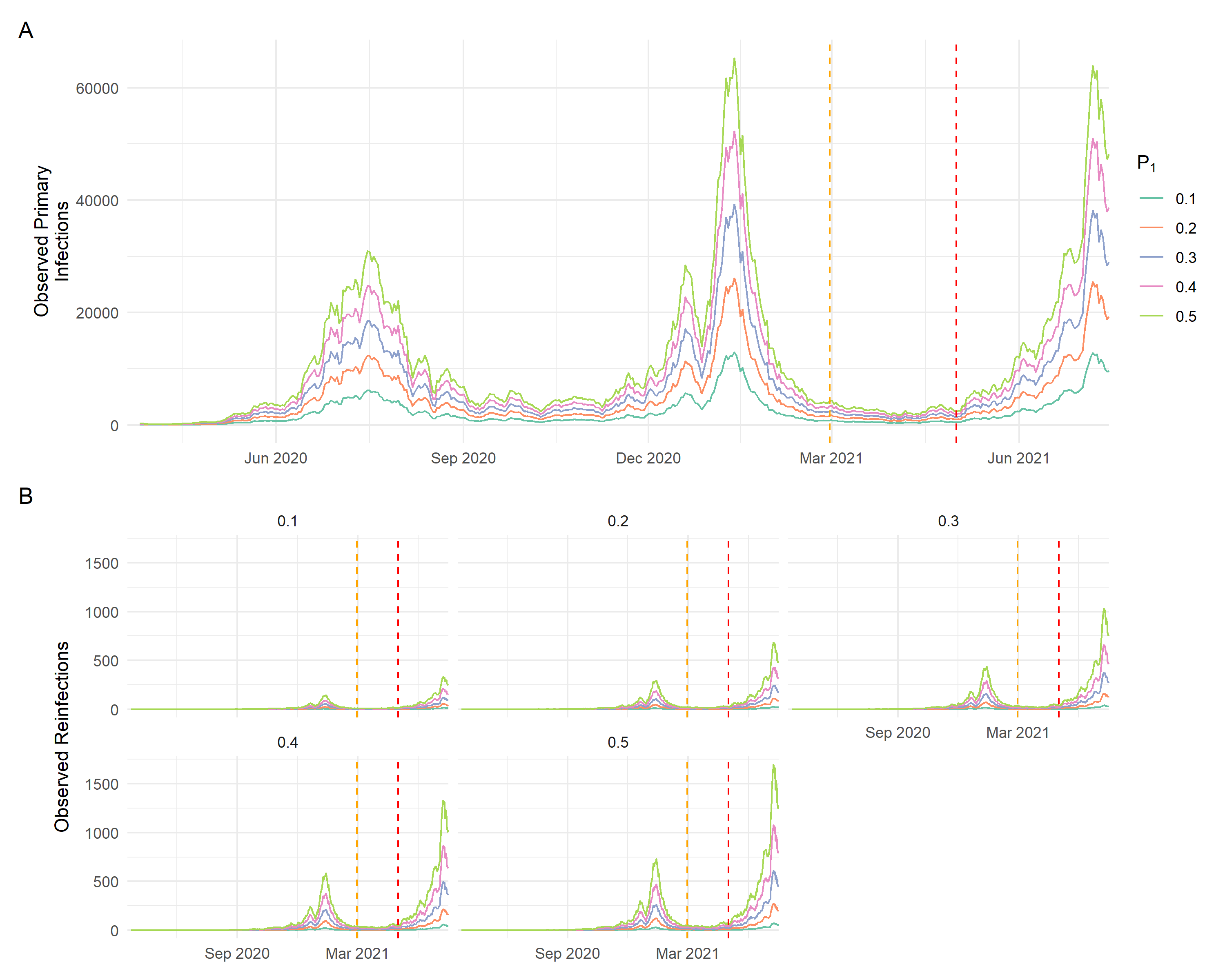

Supplement: S3 Fig — A shows the number of observed primary infections for different values of P1 and B shows the observed reinfections for different values of P2 shown at the top of each grid. Each line depicts another value ofP1. (PNG) [file pcbi.1012792.s004.png]

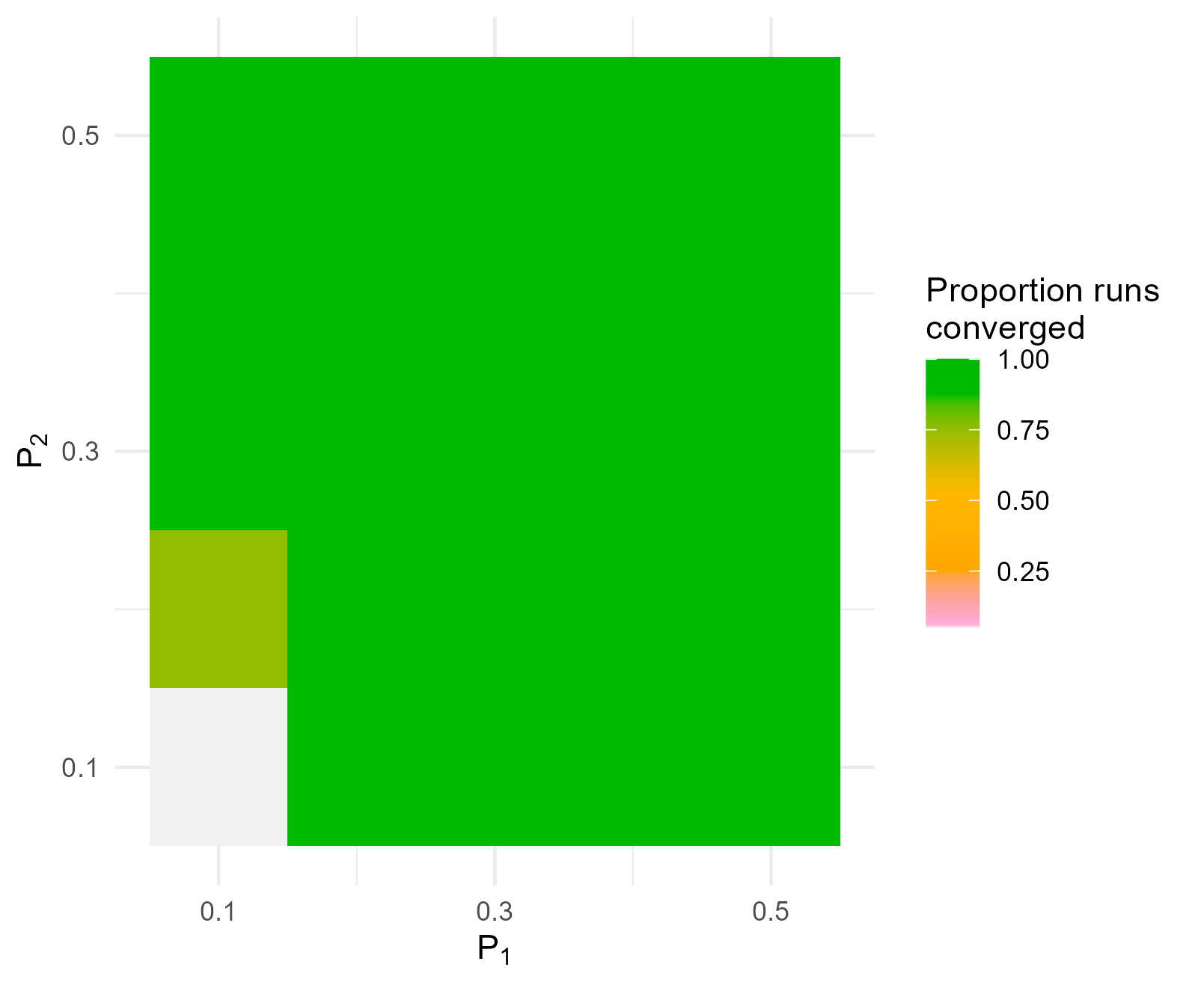

Supplement: S4 Fig — Here we introduced observation probabilities for primary infections and reinfections (P1 and P2 respectively). (PNG) [file pcbi.1012792.s005.png]

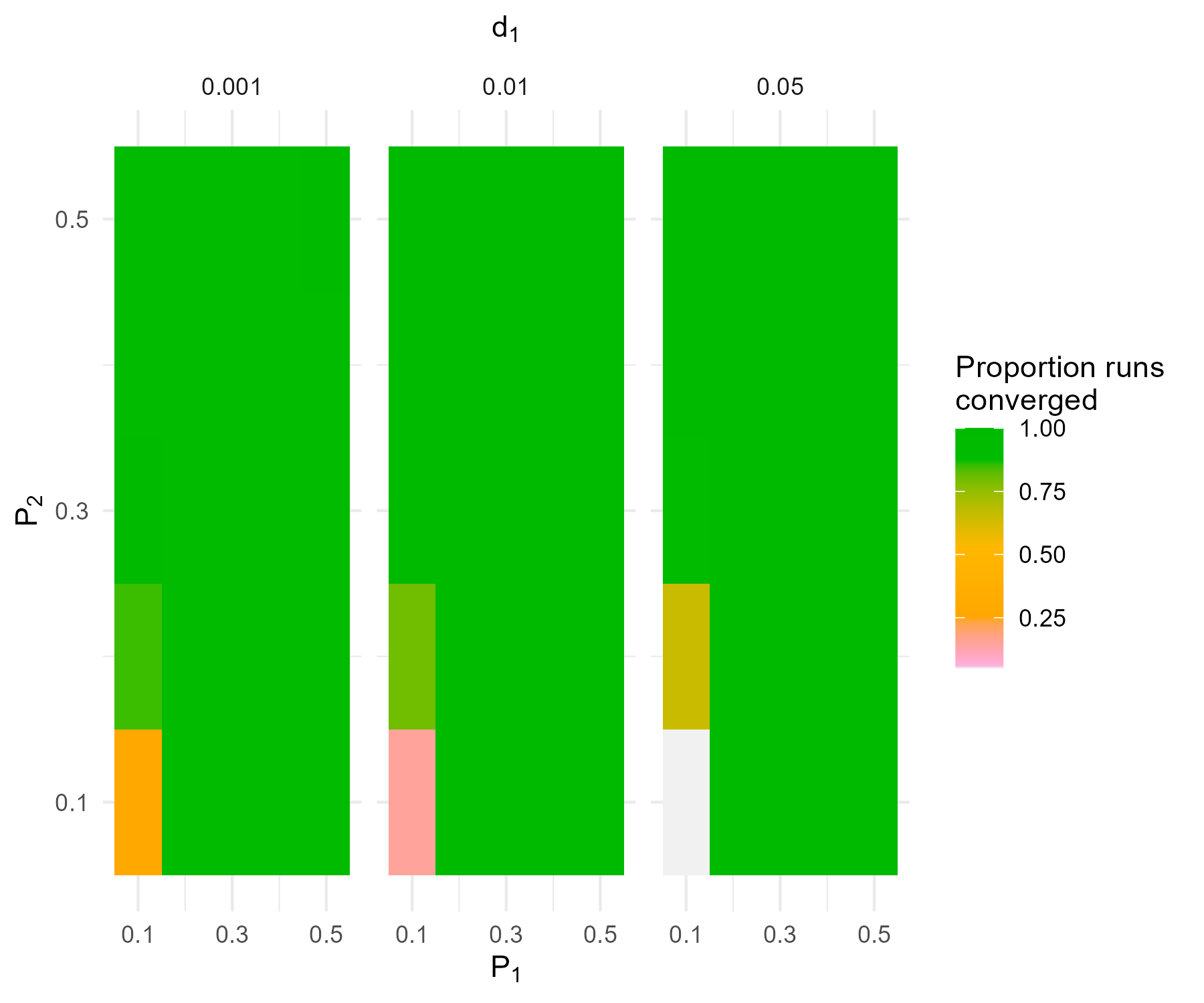

Supplement: S5 Fig — Here we added observation probabilities for primary infections, reinfections and we included mortality (P1, P2 and d1 respectively). (PNG) [file pcbi.1012792.s006.png]

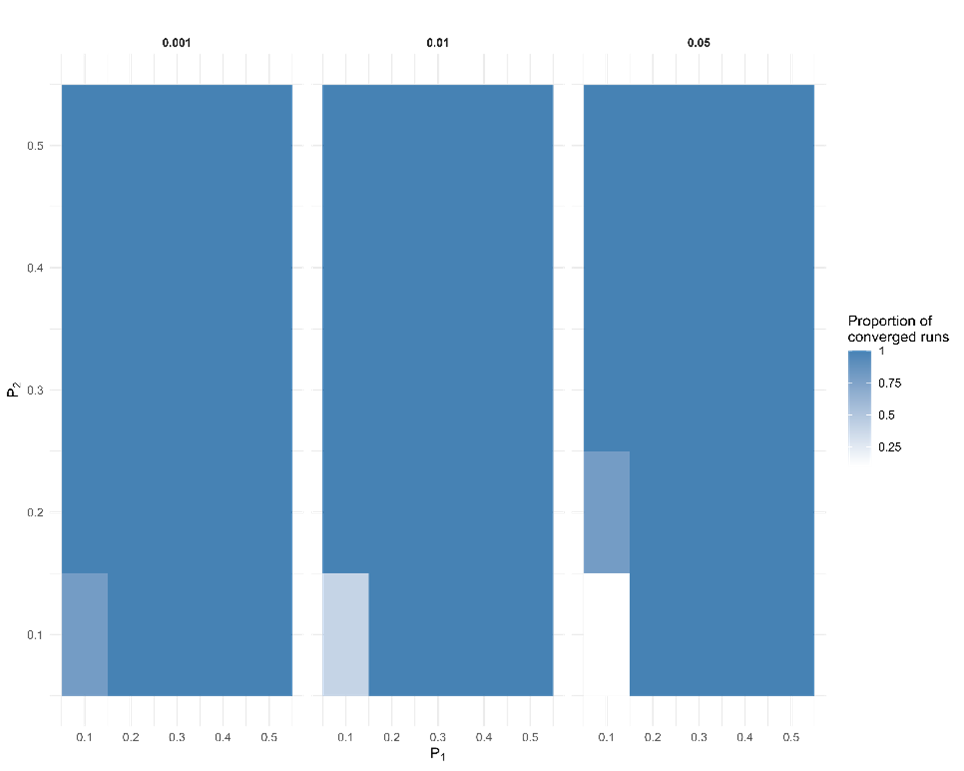

Supplement: S6 Fig — The Geweke diagnostic measures convergence by comparing the means of the first and last portions of a single Markov chain; if the Z-score is close to zero, it suggests convergence. (PNG) [file pcbi.1012792.s007.png]

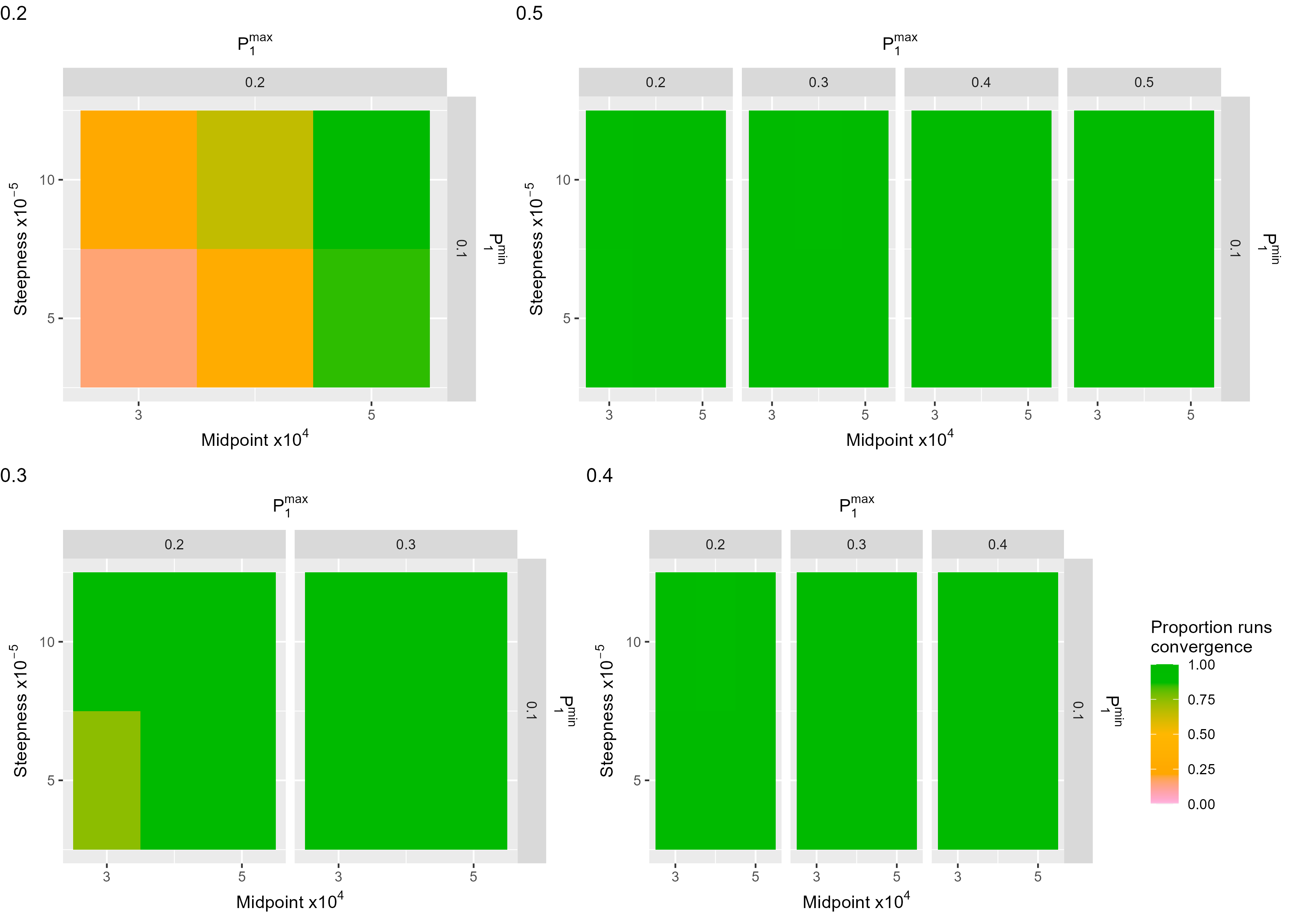

Supplement: S7 Fig — (PNG) [file pcbi.1012792.s008.png]

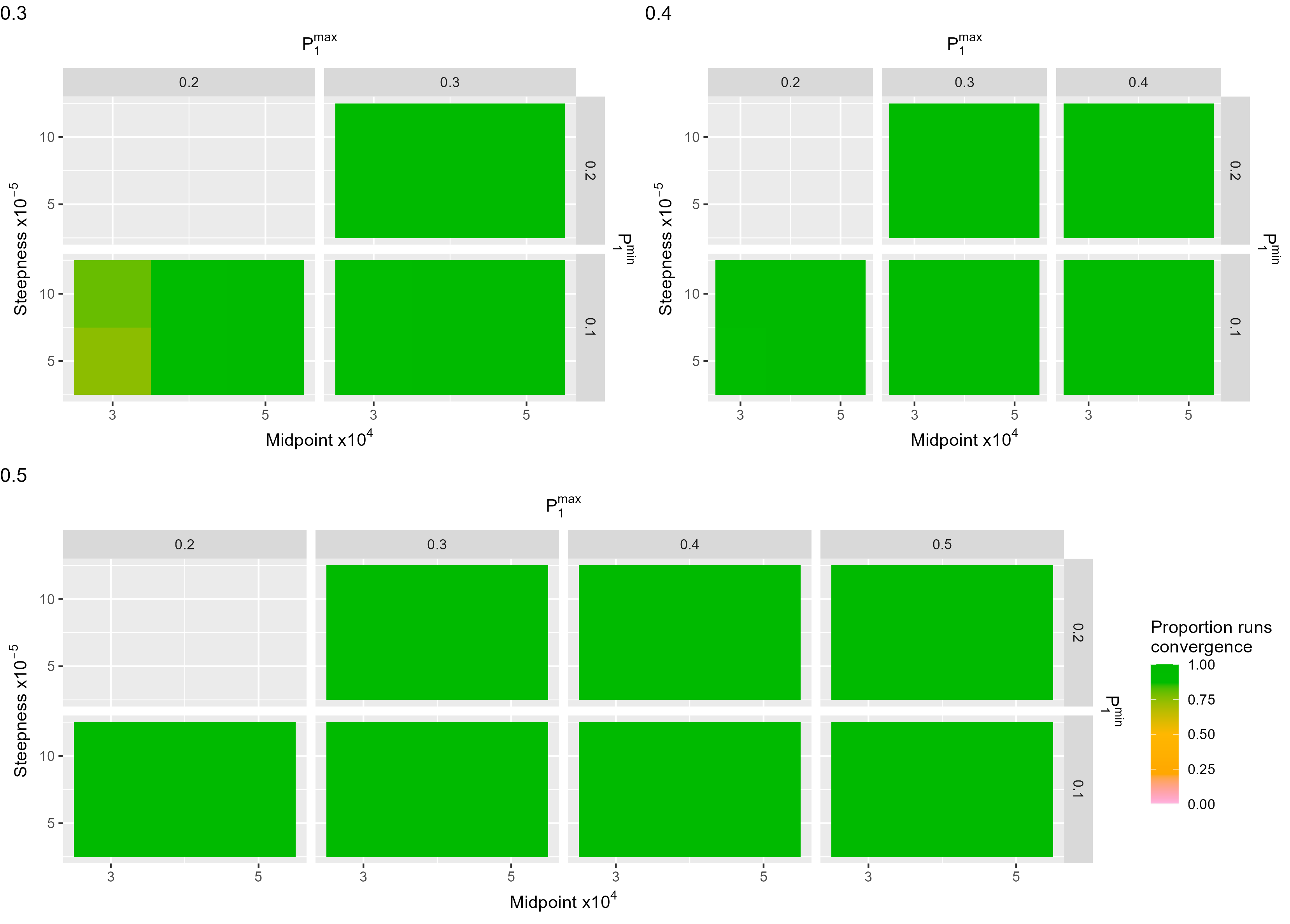

Supplement: S8 Fig — (PNG) [file pcbi.1012792.s009.png]

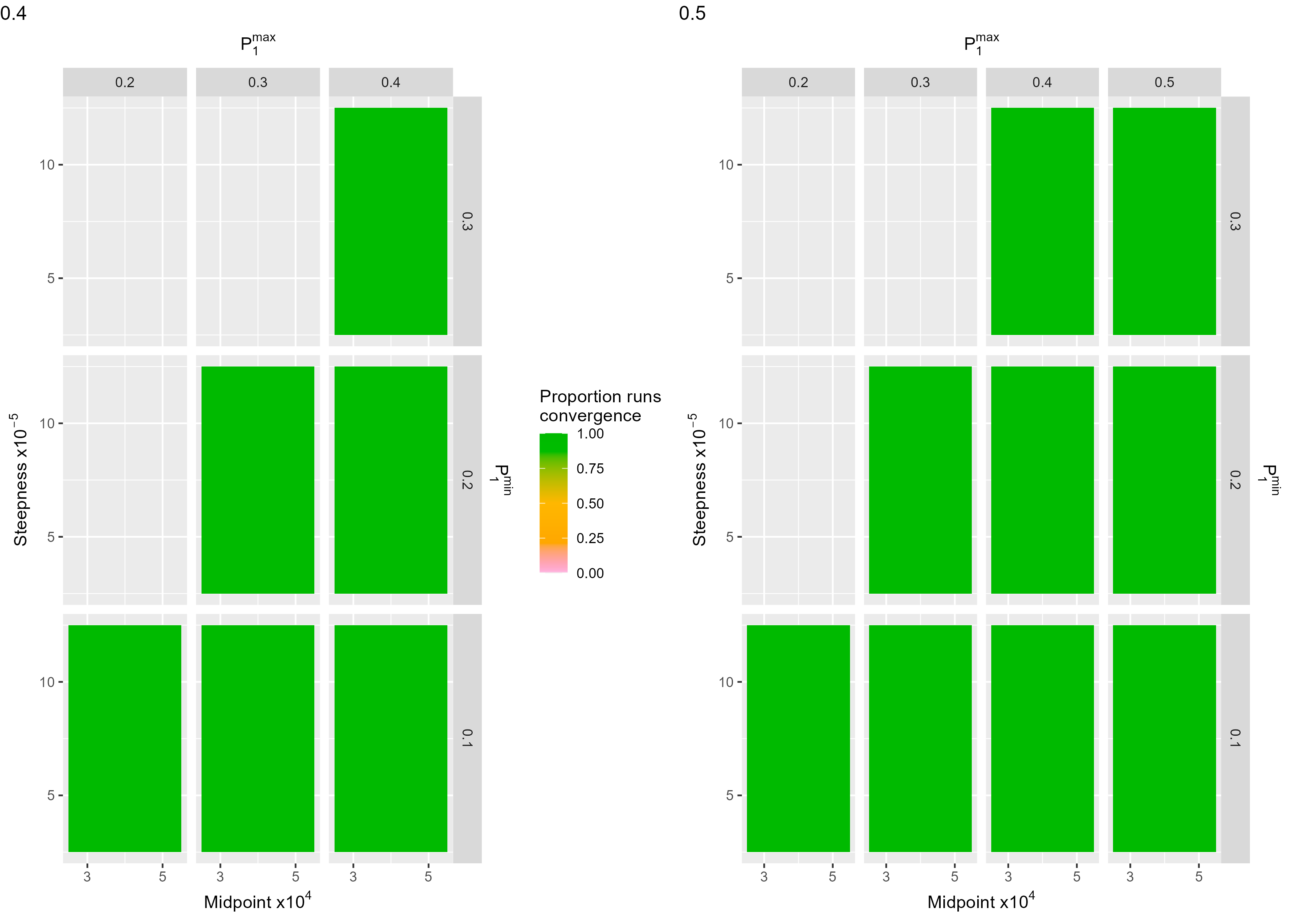

Supplement: S9 Fig — (PNG) [file pcbi.1012792.s010.png]

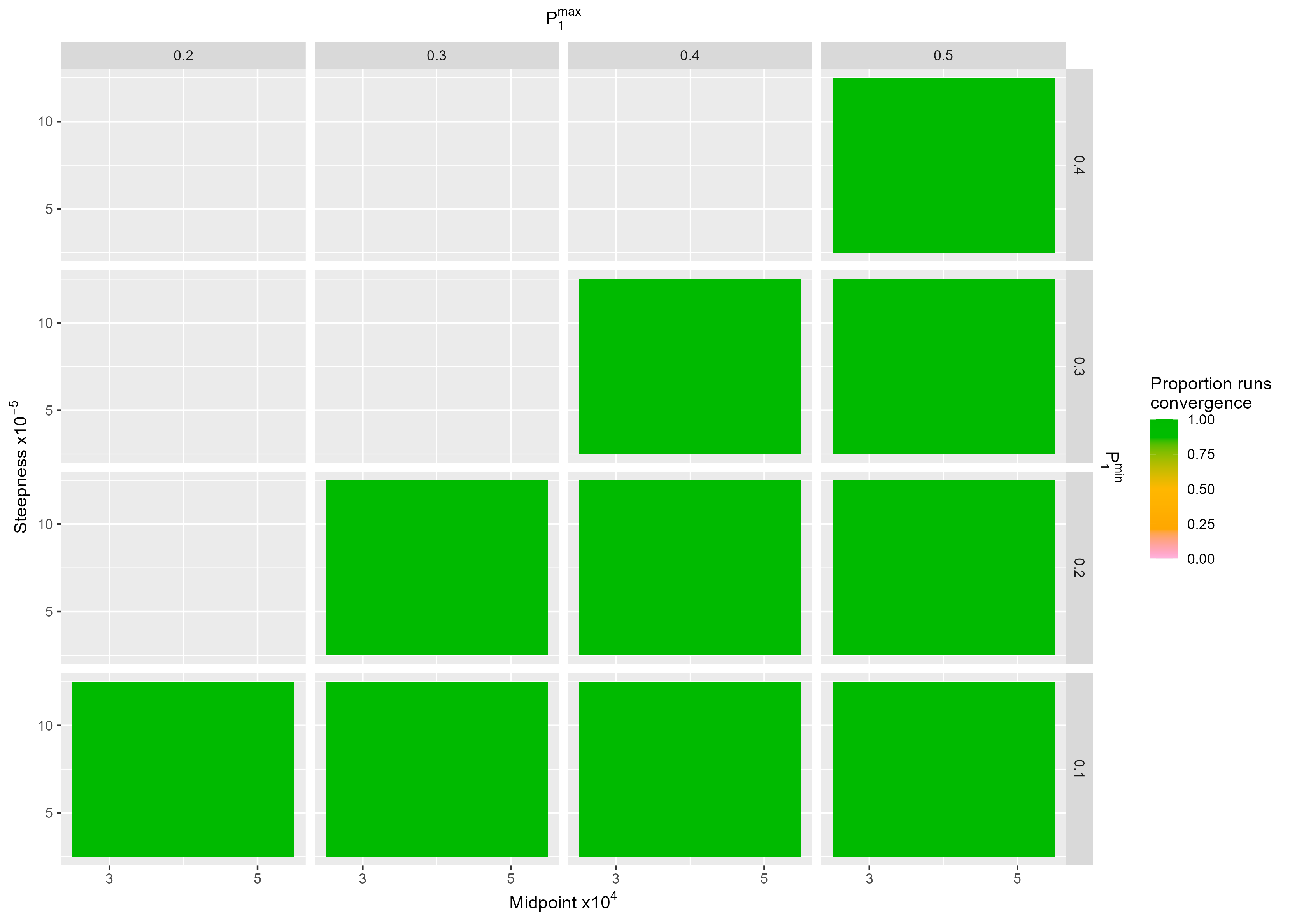

Supplement: S10 Fig — (PNG) [file pcbi.1012792.s011.png]

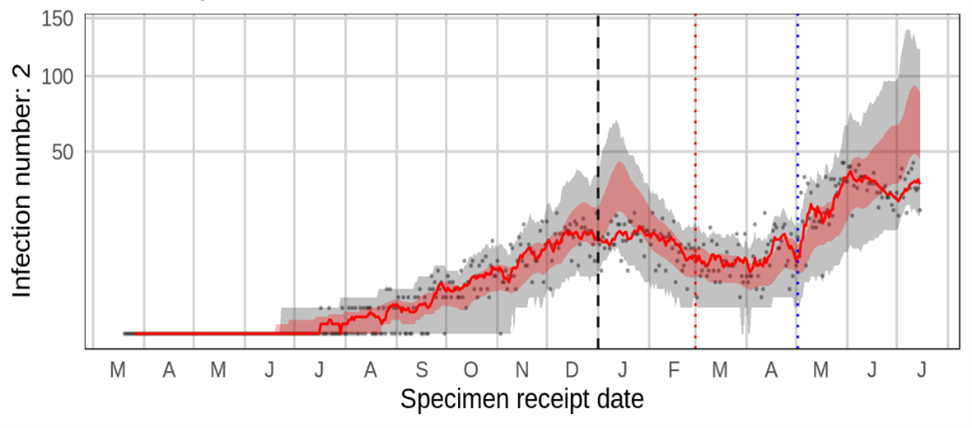

Supplement: S11 Fig — During the fitting period preceding the dotted red line, the observed reinfections (depicted by the solid red line) consistently fell below the projection interval in January. In this particular scenario, the function determining the observation probability had a low midpoint of 30,000, minimal observation probabilities for primary- and reinfections set at 0.1 and 0.4, respectively, and a low steepness factor of 0.00005. (PNG) [file pcbi.1012792.s012.png]

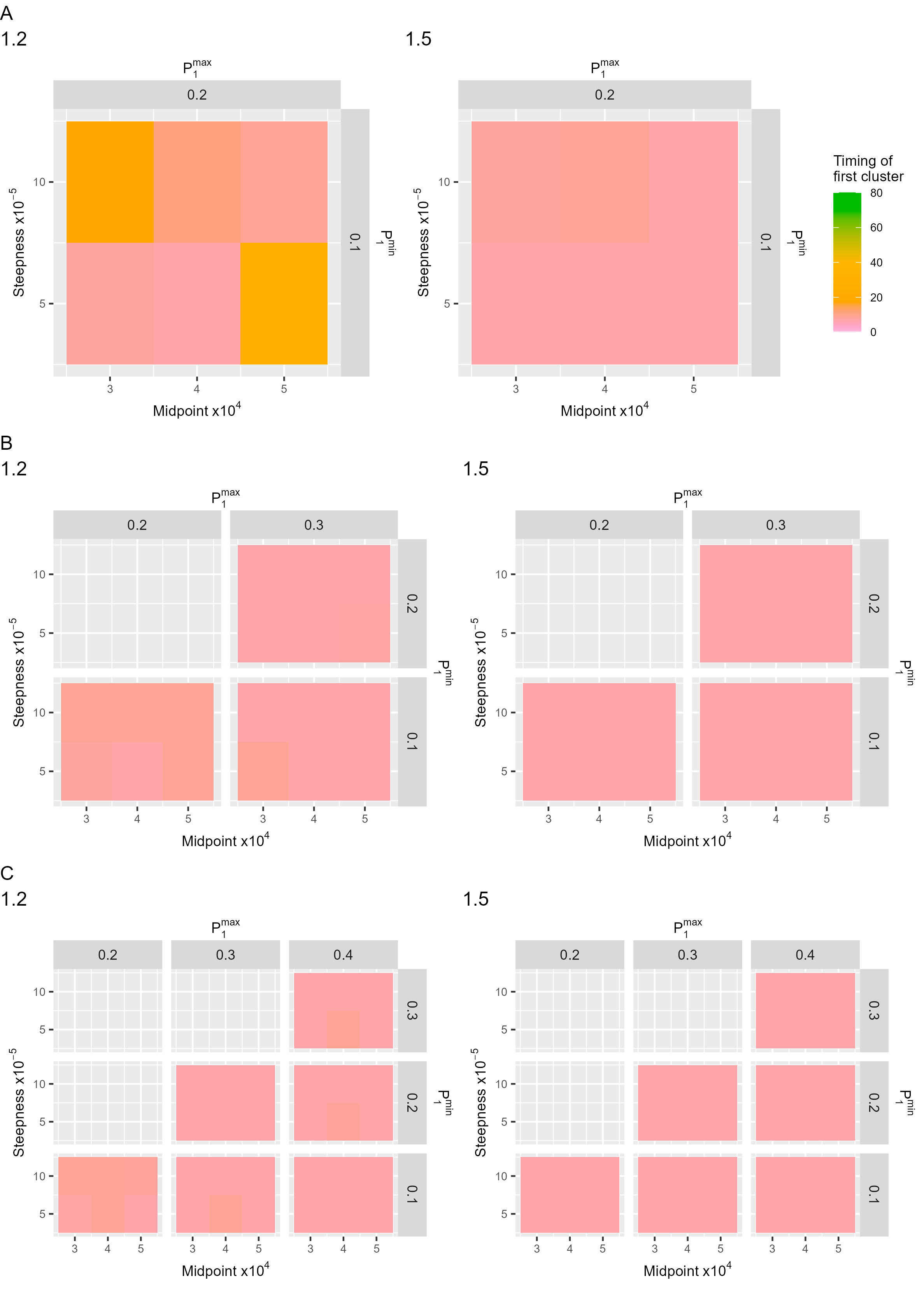

Supplement: S12 Fig — In A, the minimum and maximum observation probabilities for reinfections are 0.1 and 0.2. In B, the minimum and maximum observation probabilities for reinfections are 0.2 and 0.3. In C, the minimum and maximum observation probabilities are 0.3 and 0.4. The introduced scales (σ) are indicated at the top. (PNG) [file pcbi.1012792.s013.png]

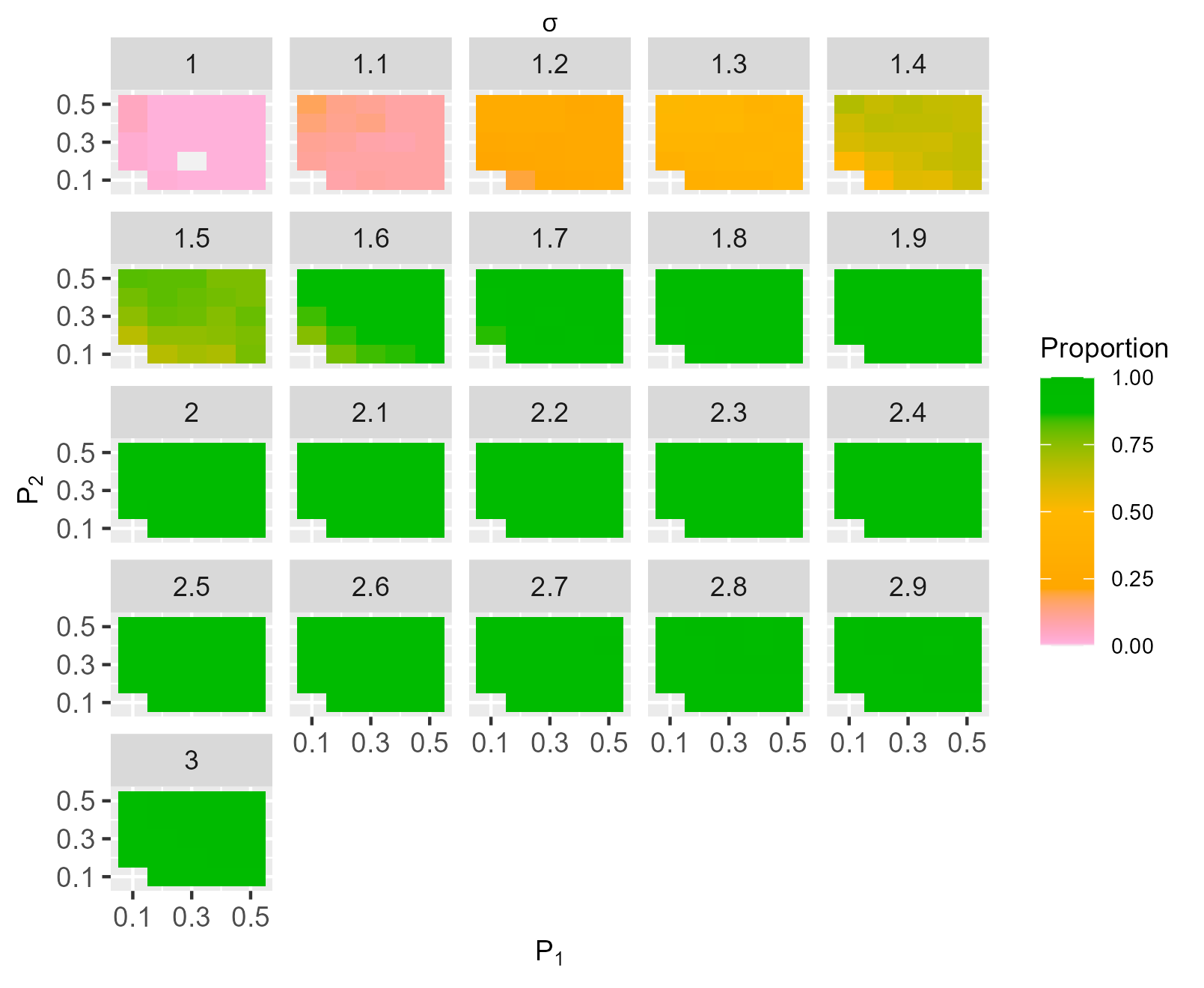

Supplement: S13 Fig — (PNG) [file pcbi.1012792.s014.png]

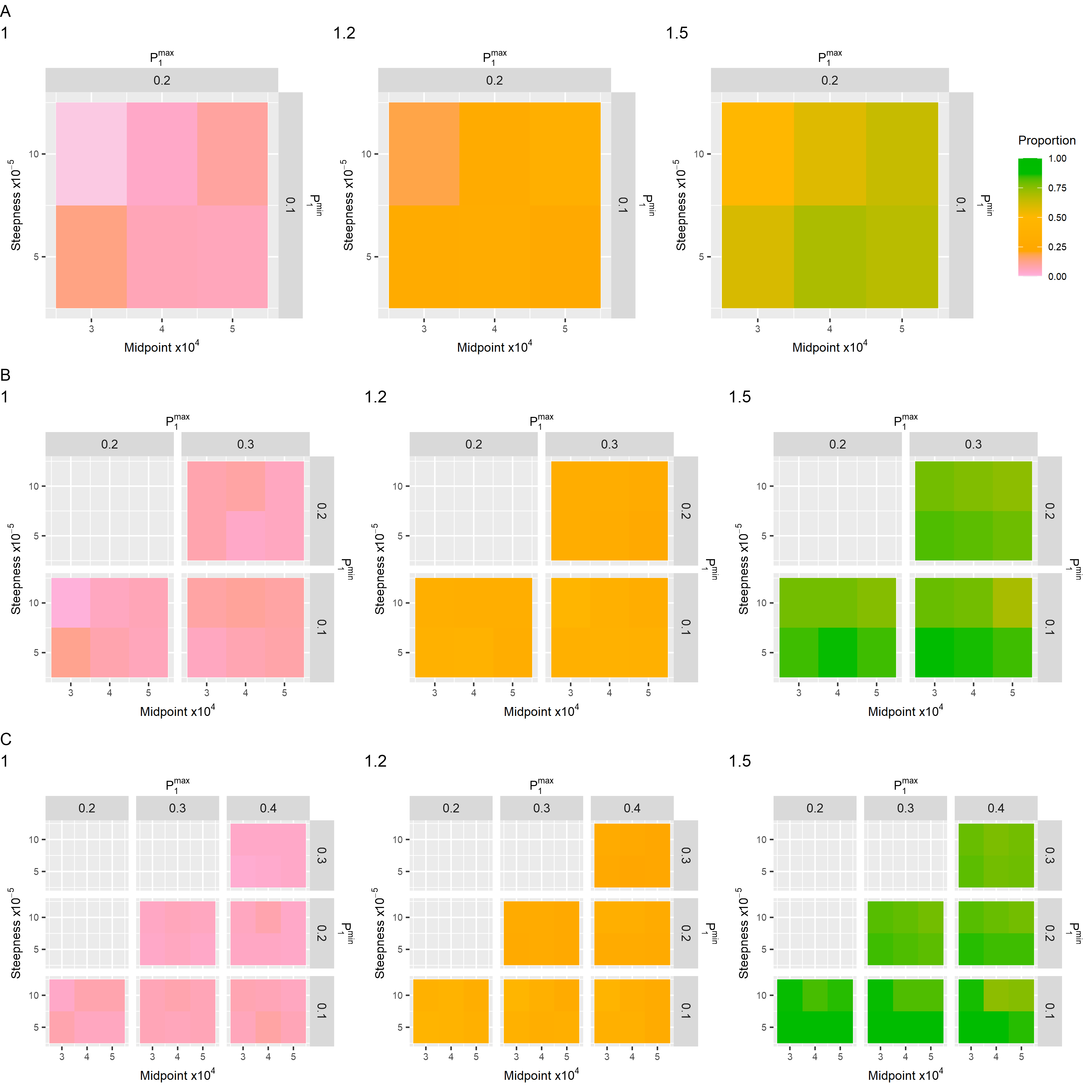

Supplement: S14 Fig — In A, the minimum and maximum observation probabilities for reinfections are 0.1 and 0.2. In B, the minimum and maximum observation probabilities for reinfections are 0.2 and 0.3. In C, the minimum and maximum observation probabilities are 0.3 and 0.4. The introduced scales (σ) are indicated at the top. (PNG) [file pcbi.1012792.s015.png]

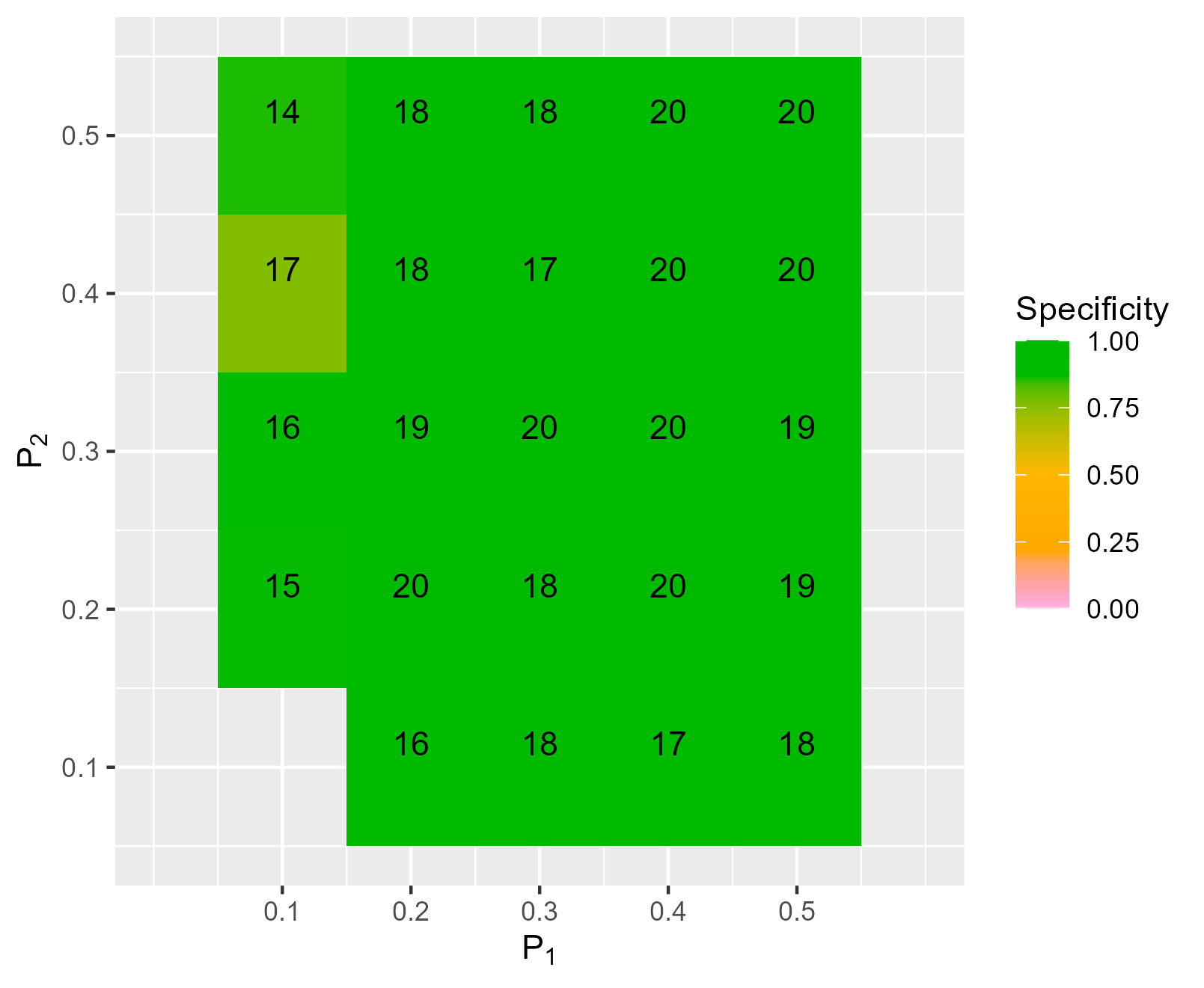

Supplement: S15 Fig — The numbers in the grid are the number of runs where both λ and κ converged and a cluster of five consecutive points above or 10 consecutive points below the projection interval during the fitting period does not exist. The specificity is measured as the number of those runs where Dfirst does not exist, i.e., no false positive detection of a change in reinfection risk was observed. (PNG) [file pcbi.1012792.s016.png]

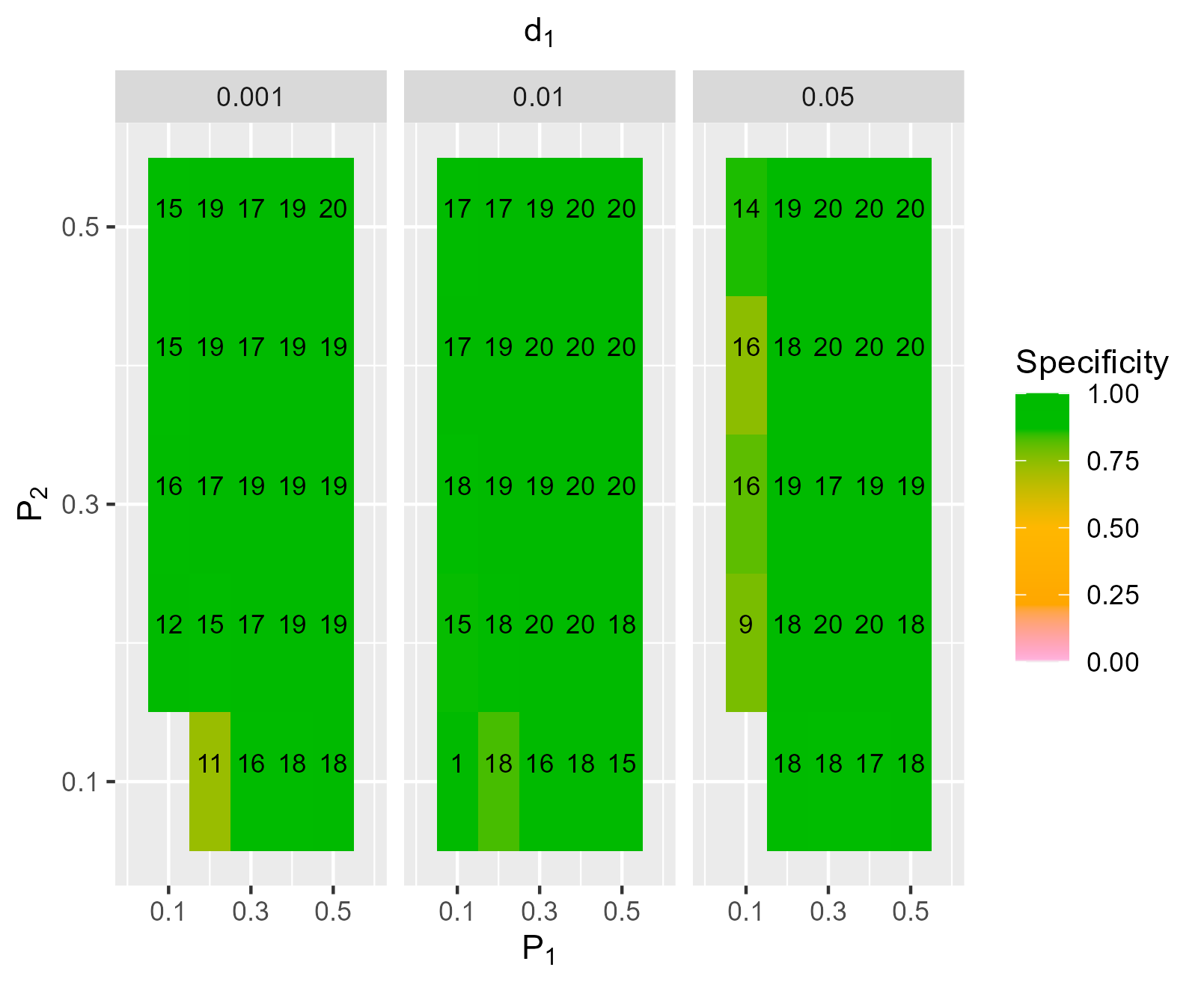

Supplement: S16 Fig — The numbers in the grid are the number of runs where both λ and κ converged and a cluster of five consecutive points above or 10 consecutive points below the projection interval during the fitting period does not exist. The specificity is measured as the number of those runs where Dfirst does not exist, i.e., no false positive detection of a change in reinfection risk was observed. (PNG) [file pcbi.1012792.s017.png]

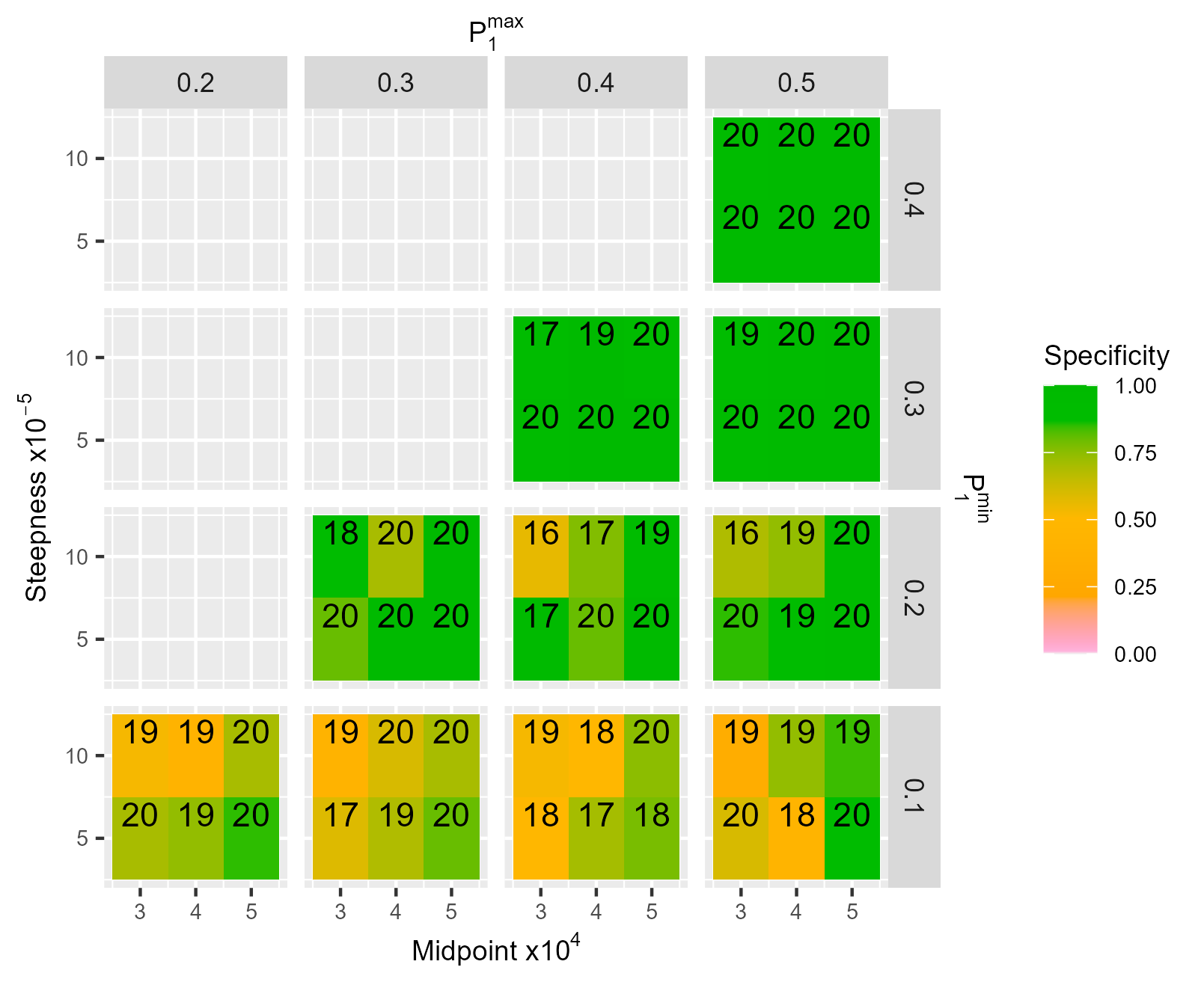

Supplement: S17 Fig — The numbers in the grid are the number of runs where both λ and κ converged and a cluster of five consecutive points above or 10 consecutive points below the projection interval during the fitting period does not exist. The specificity is measured as the number of those runs where D_first does not exist, i.e., no false positive detection of a change in reinfection risk was observed. (PNG) [file pcbi.1012792.s018.png]

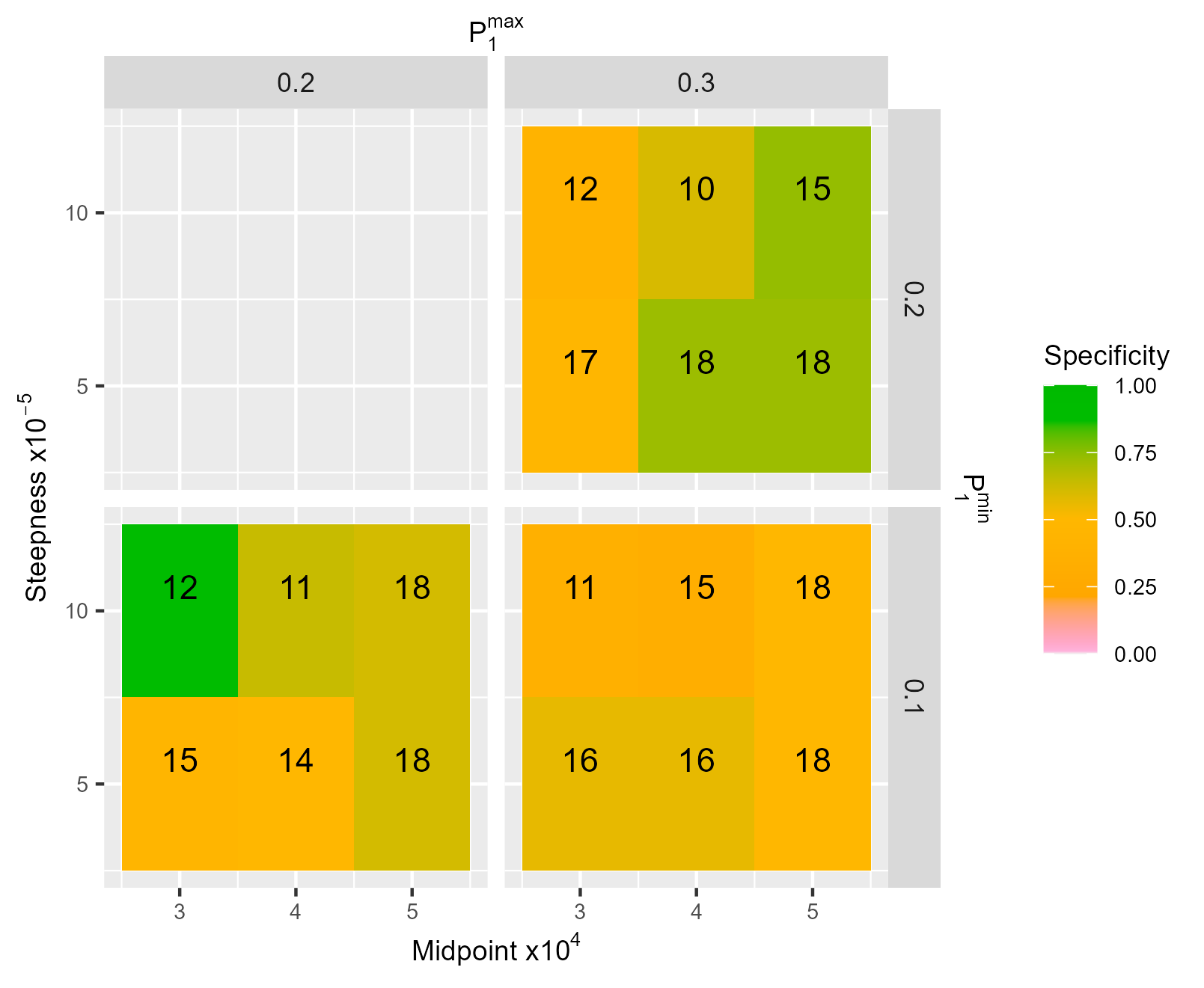

Supplement: S18 Fig — The numbers in the grid are the number of runs where both λ and κ converged and a cluster of five consecutive points above or 10 consecutive points below the projection interval during the fitting period does not exist. The specificity is measured as the number of those runs where D_first does not exist, i.e., no false positive detection of a change in reinfection risk was observed. (PNG) [file pcbi.1012792.s019.png]
